# Effectiveness of Nirmatrelvir/Ritonavir for Outpatients in the Era of Omicron, Vaccination, and Previous Infection: A Meta-analysis

**DOI:** 10.1007/s11606-026-10494-4

**Published:** 2026-05-12

**Authors:** Mark Ebell, Peter Kurotschka

**Affiliations:** 1 Department of Family Medicine, College of Human Medicine, Michigan State University, East Lansing, MI USA; 2 Department of General Practice, University Hospital Würzburg, Würzburg, Germany; 3 Athens, USA

**Keywords:** nirmatrelvir-ritonavir, paxlovid, COVID-19, SARS-CoV-2, outpatient, mortality, hospitalization, cohort

## Abstract

**Background:**

Since the benefit of nirmatrelvir-ritonavir (N-R) may have changed in contemporary patients, we assessed the effectiveness of N-R for preventing hospitalization and death among outpatients with COVID-19 in the Omicron era.

**Methods:**

This was a meta-analysis of cohort studies comparing rates of hospitalization and/or mortality in outpatients treated with N-R compared with untreated patients. Analysis was limited to studies conducted since December 2021 that performed an adjusted multivariate analysis. Quality was assessed using the Newcastle-Ottawa Scale. Summary estimates of adjusted relative risks (aRR) with 95% confidence intervals (CI) and prediction intervals (PI) were calculated overall and for prespecified subgroups. Heterogeneity was summarized visually and with τ and 95% PIs. Absolute effects were estimated by applying pooled aRRs to baseline risks to obtain absolute risk reductions (ARRs) and numbers needed to treat (NNTs).

**Results:**

Forty-seven studies (10,791,211 patients) were included. Pooled aRRs were 0.54 (95% CI, 0.43–0.68) for all-cause hospitalization and 0.45 (0.36–0.56) for COVID-19 hospitalization. Pooled RRs were 0.30 (0.23–0.39) for all-cause mortality and 0.43 (0.32–0.59) for COVID-19 mortality. PIs were < 1.0 for COVID-19 hospitalization (0.21–0.96), all-cause mortality (0.11–0.83), and any mortality (0.13–0.88), indicating likely benefit in future studies in similar settings. Subgroup analyses showed larger effects earlier in the Omicron period for hospitalization (RR 0.46 vs 0.68; *p* = 0.0049) and the composite outcome (0.45 vs 0.68; *p* = 0.0078), and a smaller mortality reduction among immunocompromised patients (RR 0.26 vs 0.11; *p* = 0.034). The estimated NNT to prevent a COVID-19 hospitalization for patients at low risk (0.16%), moderate risk (2.2%), and high risk (8.9%) of hospitalization based on a validated risk score were 1148, 84, and 20 respectively.

**Discussion:**

N-R is associated with reduced hospitalization and death. Absolute risk reductions of hospitalization are small in low-risk patients but clinically meaningful in moderate- and high-risk patients.

**Supplementary Information:**

The online version contains supplementary material available at https://doi.org/10.1007/s11606-026-10494-4.

## INTRODUCTION

Nirmatrelvir-ritonavir (N-R, marketed as Paxlovid) was initially evaluated in 2246 high-risk patients who were largely exposed to the ancestral and alpha variants of SARS-CoV-2 and prior to the development of a vaccine. In that study, N-R caused a large and clinically important reduction in hospitalizations and deaths (0.8% vs 6.3%, *p*< 0.001)^1^. A subsequent randomized trial in 1296 average-risk patients (some of whom were vaccinated) during the delta wave of the pandemic found a numeric reduction in hospitalizations (0.8% vs 1.6%) that was not statistically significant^2^.

The current environment differs from that in which N-R was studied in randomized trials. It is characterized by the omicron variant, high rates of immunization in most countries, and high rates of previous infection. As a result, hospitalization rates for outpatients are much lower than during the first phase of the pandemic. For example, in published reports, the rate of hospitalization in untreated patients was 6.3% in 2020^1^, 1.6% in 2021 to 2022^2^, and 1.0% in 2022 to 2023^3^. One result of lower hospitalization rates is that the absolute risk reduction for hospitalization is likely to be lower, increasing the number needed to treat.

Previous systematic reviews have evaluated the efficacy of N-R in observational studies^4–8^. The largest and most recent included 22 studies that collected data through September 2023^5^. The authors concluded that N-R remained effective with a relative risk for the composite of hospitalization or death of 0.62 (95% CI 0.55 to 0.70). However, limitations of previous meta-analyses include an incomplete or dated search, inclusion of studies in hospitalized patients, and inclusion of older studies during earlier phases of the pandemic. Our goal is to perform an updated meta-analysis of cohort studies comparing outpatients treated with N-R with untreated patients, including only studies that adjusted for key potential confounders, gathered data during the omicron phase of the pandemic, and reported 30-day hospitalization and/or mortality as an outcome.

## METHODS

This was a systematic review and meta-analysis of published cohort studies comparing patients treated with nirmatrelvir-ritonavir with an untreated comparison group. As the study was limited to published reports, we did not seek ethical approval. We adhered to the Preferred Reporting Items for Systematic Reviews and Meta-Analyses (PRISMA) guidelines for reporting^9^. The protocol was registered with PROSPERO on June 23, 2025 (CRD420251078971). The study was unfunded.

### Search Strategy

Using multiple terms for ‘nirmatrelvir-ritonavir’ *and* ‘cohort study’ *and* (‘hospitalization’ *or* ‘mortality’) we searched Medline via PubMed and Cochrane Central until July 6, 2025, and updated the searches on October 6, 2025. The reference lists of relevant systematic reviews^4–8,11–16^ were also searched. The full search strategy is available in Appendix Text 1.

### Study Selection and Inclusion Criteria

For screening, titles and abstracts of retrieved studies were imported into Rayyan^17^. Potentially eligible studies were reviewed in full against the predefined inclusion criteria. Either stage of study selection was done independently by the two review authors, and any disagreement was solved through discussion.

We included studies of adult and adolescent (aged 12 or older) outpatients diagnosed with COVID-19 by PCR who were not initially hospitalized and that compared N-R with untreated controls and reported at least one outcome of hospitalization, death, or the composite of hospitalization or death. The omicron variant was first reported in November 2021, and by January 2022, it was the dominant variant worldwide^18^. Included studies were therefore limited to those that began gathering data no sooner than December 2021. Only studies that performed an adjusted analysis (e.g., propensity score matching, logistic regression, Cox regression) were included. Studies comparing two or more antivirals without an untreated control group, case-control studies, non-peer-reviewed reports, and studies of a special population (e.g., pregnant persons or patients with liver disease, transplant, cancer, chronic kidney disease) were excluded.

### Data Abstraction and Preparation

We independently abstracted data for analysis on Google spreadsheets with any discrepancies resolved through discussion. Study characteristics including country, timeframe, method of statistical adjustment, covariates, and demographics were extracted by one author (ME) and reviewed independently by the other author (PK) for correctness. Data used for the meta-analysis (adjusted relative risk, odds ratio, or hazard ratio with 95% confidence intervals) was reviewed and abstracted in parallel by both authors with discrepancies reconciled by consensus discussion.

Mortality was classified as 30-day all-cause or 30-day COVID-related, as was hospitalization. The composite outcome of hospitalization or mortality could use any definition. As prespecified, if results were not significantly different based on *p* > 0.05 for all-cause versus COVID-specific outcomes, then those outcomes were combined into any hospitalization, any mortality, and any hospitalization or mortality for subgroup analyses.

Prespecified subgroup analyses included the following: (1) propensity score matching (PSM) versus other approaches to adjustment for confounders; (2) vaccination status; (3) age group; (4) immunocompromise; and (5) low versus high baseline risk of the study outcome. We also prespecified an analysis of earlier versus later studies during the omicron phase of the pandemic, since we anticipated that patients would have more boosters and previous infections later in the pandemic.

For these analyses, we combined comparable subgroups from the primary studies into single variables, as detailed in Appendix Table 1. Briefly, patients identified as having any degree of immunocompromise were classified as immunocompromised (see Appendix Table 5 for study-specific definitions), and age groups were combined if similar. Vaccination was classified as no vaccination, primary series, primary series plus booster, or primary series or primary series plus booster. Data from studies with other classifications such as 1 mRNA vaccination or one or more vaccinations were excluded as these groupings could include patients with and without vaccine-based immunity. If a study only stated that patients were vaccinated without further detail, it was classified as “Primary series or primary series + booster”.

Low- and high-risk groups were identified after reviewing the distribution of mortality and hospitalization rates post hoc in the untreated control groups. Studies with a mortality rate less than 0.5% in the control group were classified as low risk. Studies not reporting mortality were classified as low risk if the hospitalization rate in the control group was less than 4.0%.

### Assessment of Study Quality

The Newcastle-Ottawa Quality Assessment Scale (NOS) for cohort studies was used to assess the risk of bias in included studies with regard to three domains: the cohorts’ selection, the comparability of cohorts, and the adequacy of outcome ascertainment and follow-up (Appendix Table 3)^19^. One author (ME) assessed the risk of bias for all of the studies. Judgements for each study were then reviewed by the other author for accuracy (PK). NOS ratings for each study were then mapped to the Agency for Healthcare Research and Quality (AHRQ) categories of good quality (9 stars at NOS rating), fair quality (7–8 stars), or poor quality (< 7 stars).

For the outcomes any hospitalization and any mortality, we conducted a prespecified sensitivity analysis restricted to studies judged good quality to evaluate the robustness of the findings to risk of bias. Immortal time bias occurs when there is a delay between the start of follow-up and when nirmatrelvir-ritonavir is started in the treatment group, a period that the participant must survive (hence immortal) to be included in the treatment group. Strategies to avoid it include using time-dependent covariates and only beginning outcome assessment at a point when all patients would have presumably started treatment such as day 2 after the index clinic visit. We also compared the main results for studies that did and did not explicitly address immortal time bias.

### Statistical Analysis

Meta-analyses of adjusted risk ratios were performed using random effects models with a restricted maximum likelihood (REML) estimator to calculate pooled effect estimates alongside 95% confidence intervals (95% CIs). REML was chosen because it is preferred in situations where there is a rare binary outcome and when there is a wide range of study sizes^20^. Studies reported events as relative risks (risk ratios), odds ratios, and hazard ratios. Since the incidence of deaths and hospitalizations was low and the follow-up period was short, standard methods were used to estimate relative risks from odds ratios and hazard ratios^19^. Where the hospitalization rate was not reported by a study, we conservatively used 5% for hospitalization in these calculations^21,22^.

To translate pooled relative risks into absolute terms, we applied pooled RRs to externally derived baseline risks. Baseline risks of hospitalization for outpatients were taken from the Lehigh Outpatient COVID-19 Hospitalization (LOCH) risk score strata (low 0.16%, moderate 2.2%, high 8.9%) which was validated during the omicron phase^3^. Treated risk was calculated as *RR* × *baseline risk* (using the pooled RR for COVID-19 hospitalization), the absolute risk reduction (ARR) as *baseline − treated risk*, and the number needed to treat (NNT) as *1/ARR*. Values were rounded to one decimal place for ARR (%) and to the nearest integer for NNT. These estimates assume a multiplicative treatment effect that is approximately constant across baseline-risk strata, consistent with the observed stability of RRs across control-group risks.

Heterogeneity was estimated using tau and 95% prediction intervals; the latter incorporate both within- and between-study heterogeneity. While *I*^2^is often used to measure heterogeneity in meta-analyses, it is unreliable with very large studies that have small variances and narrow confidence intervals^23^. Both confidence and prediction intervals were therefore calculated for overall and subgroup outcomes^24^.

We constructed funnel plots of study log–RRs versus their standard errors for the outcomes of any mortality, any hospitalization, and the composite hospitalization and mortality. We assessed them visually for small-study publication bias (asymmetry around the pooled effect). Because few small studies contributed to each outcome, we did not perform formal asymmetry tests.

All analyses were performed using R version 4.5.0 (2025-04−11) and R Studio version 2024.12.1 using the meta package^25,26^.

## RESULTS

**Figure 1:**
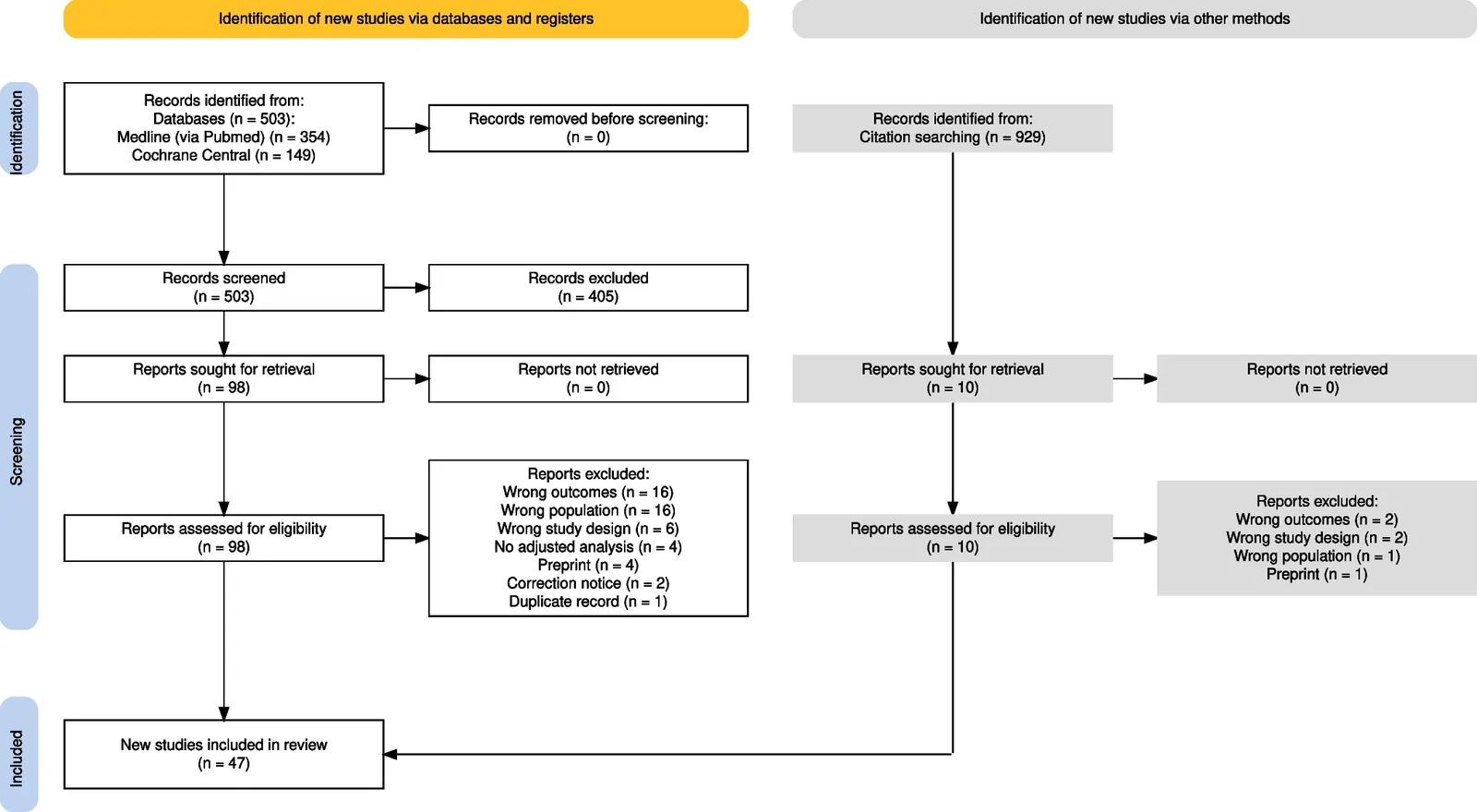
PRISMA flow diagram of included studies.

We screened 1432 records in total and reviewed 108 of them in full. We finally included 47 cohort studies with a total of 10,791,211 patients. The PRISMA flow diagram summarizing the search results is shown in Fig. 1^27^.

### Study Characteristics

Twenty studies were set in the USA and Canada, 16 in Asia, 5 in Europe, 3 in multiple countries, and one each in Mexico, Israel, and Dubai, which reported that the mean age ranged from 38 to 83 years, and the median age from 66 to 82 years. The percentage of patients that were identified as female ranged from 12 to 67%. The rate of hospitalization in the control groups ranged from 0.47%^28^to 67.5%^29^, and of mortality from 0.05%^30^to 5.61%^31^. If one excludes two outlier studies set in Hong Kong^29,32^, the hospitalization rates ranged from 0.47 to 11.9%. Studies ranged in size from 213 patients to 2,300,131 patients. Table 1 summarizes study characteristics; additional characteristics are shown in Appendix Table 2.

**Table 1:** Study Characteristics

**Author, year**	**Country**	**Population and setting**	**Age**	**% female**	**Events in control group (%)**			**Dates data collected**	**Number of patients**	
					**Hosp or death**	**Hosp**	**Death**		**Nirm/rit**	**Control**
Aggarwal, 2023^43^	USA	Outpatients with COVID-19 or prescription for nirmatrelvir/ritonavir	Tx: 32% 65 + Cx: 21% 65 +	58%	NR	1.4%	0.16%	3/26/2022 to 8/25/2022	7168	9361
Aggarwal, 2024^44^	USA	Outpatients with COVID-19 or prescription for nirmatrelvir/ritonavir	65+: 44%	58%	NR	2.0%	NR	4/3/2022 to 11/12/2022	12,061	10,031
Al-Obaidi, 2023^45^	USA	Outpatients with mild to moderate COVID; excluded if ED	Mean: 58	40%	NR	2.1%	0.10%	6/1/2022 to 9/24/2022	5754	5754
Arbel, 2022^33^	Israel	Outpatients 40 and older with COVID-19 and at least one risk factor for severe disease	Mean 60	60%	NR	1.0%	NR	1/9/2022 to 3/31/2022	3902	105,352
Bajema, 2023^34^	USA	Outpatient adult military veterans with at least 1 risk factor for severe disease and a positive COVID-19 test	Median: 66	14%	3.41%	3.0%	0.55%	1/1/2022 to 7/31/2022	9607	9607
Bhatia, 2025^35^	USA	Outpatients with COVID-19 and at least one risk factor for severe disease	Tx: 32% 50 to 64, 40% 65+ Cx: 28% 50 to 64, 31% 65+	63%	NR	2.4%	0.26%	4/1/2022 to 8/28/2023	206,562	497,085
Butt, 2024^46^	USA	Non-hospitalized adult military veterans diagnosed with a first COVID-19 infection and if treated within 3 days	Mean 63	15%	NR	5.8%	NR	1/1/2022 to 2/25/2023	7615	7615
Cegolon, 2023^47^	Italy	Outpatients at high risk for complications diagnosed with COVID-19	Mean: 68	51%	NR	7.2%	NR	2/1/2022 to 5/31/2022	102	111
Cullen, 2024^36^	USA	Non-hospitalized persons 12 and older with COVID-19 and with at least 1 risk factor for severe infection	Mean: 59	60%	NR	5.9%	NR	12/22/2021 to 7/20/2022	12,439	36,490
Dormuth, 2023^37^	Canada	4 groups of outpatients with COVID and varying degrees of immunocompromise	Group: 1: Median 61 2: Median 63 3: Median 73 4: Median 79	Group: 1: 64% 2: 61% 3: 52% 4: 47%	NR	3.4%	NR	2/1/2022 to 2/3/2023	Group: 1: 280 2: 1314 3: 1050 4: 789	Group: 1: 280 2: 1314 3: 1050 4: 789
Dryden-Peterson 2023^30^	USA	Outpatients 50 years and older diagnosed with COVID-19	Tx: 54% 50 to 64, 46% 65+ Cx: 55% 50 to 64, 44% 65+	40%	0.97%	NR	NR	1/1/2022 to 7/17/2022	12,541	32,010
Evans 2023^48^	Wales (UK)	High risk outpatients (immunocompromised, CKD, liver disease, sickle cell, post-transplant, HIV)	Tx: Mean 53 (all drugs) Cx: Mean 57	Tx: 61% Cx: 54%	10.94%	NR	NR	12/16/2021 to 4/22/2022	602	4973
Faust, 2023^49^	USA	Vaccinated outpatients 18 to 50 years with COVID-19 diagnosis	Mean: 38	67%	NR	1.7%	0.39%	12/1/2021 to 7/30/2022	2547	2547
Ganatra, 2023^50^	USA	Vaccinated outpatients 18 and older with COVID-19 diagnosis	Mean: 58	63%	NR	2.0%	0.88%	12/1/2021 to 4/18/2022	1130	1130
Hansen, 2023^51^	USA	Outpatients diagnosed with COVID-19 and at least one risk factor for severe disease	Tx: Mean 58 Cx: Mean 50	63%	NR	0.9%	0.16%	12/2021 to 2/2023	98,060	914,850
Henderson, 2024^52^	USA	Adults diagnosed with COVID-19	Mean: 47	61%	NR	0.5%	NR	1/3/2022 to 8/15/2022	4948	39,723
Hsu CK 2024^53^	USA	Outpatients with COVID-19 and at least one risk factor for severe disease	Mean: 62	60%	3.79%	3.4%	0.40%	2/1/2023 to 8/31/2023	6,358	6,358
Huh, 2024^54^	Republic of Korea	Outpatients 60 years and older diagnosed with COVID-19	Mean: 72	59%	NR	NR	0.35%	1/1/2022 to 12/31/2022	957,036	957,036
Jorda, 2025^55^	Austria	Outpatients with COVID-19 and with at least one risk factor for severe COVID	Tx: 88% 60+ Cx: 61% 60+	44%	NR	1.1%	0.13%	1/1/2022 to 5/30/2023	12,166	90,481
Kabore, 2023^56^	Canada	Outpatients diagnosed with COVID-19 and with at least 1 risk factor for severe disease	60+ years: 56%	58%	NR	3.4%	NR	3/15/2022 to 10/15/2022	8402	8402
Kim, 2023^57^	Republic of Korea	Outpatients diagnosed with COVID-19 and with at least 1 risk factor for severe disease	Tx: 89% 60+ years Cx: 74% 60+ years	58%	NR	NR	0.15%	7/1/2022 to 11/30/2022	420,996	1,515,959
Kim, 2024^58^	Republic of Korea	Outpatients 12 and older diagnosed with COVID and with at least 1 risk factor for severe infection	Mean: 69	60%	NR	NR	0.27%	7/24/2022 to 3/31/2023	62,987	251,948
Kuo, 2025^28^	Taiwan	Outpatients 12 and older diagnosed with COVID and with at least 1 risk factor for severe infection	Tx: 75.5% 65+ years Cx: 21.4% 65+ years	58%	0.5	0.50%	0.20%	1/1/2022 to 12/1/2022	530,807	1,769,324
Kwok, 2023^59^	Hong Kong	Unvaccinated outpatient adults with COVID and chronic respiratory disease (COPD, asthma or bronchiectasis)	Tx: Median 79 Cx: Median 82	Tx: 36% Cx: 26%	NR	9.6%	5.03%	2/26/2022 to 8/26/2022	302	2387
Lewnard, 2023^60^	USA	Outpatients 12 and older diagnosed with COVID	Tx: 44% 50 to 69, 31.2% 70+ Cx: 33% 50 to 69, 10.5% 70+	56%	NR	0.56%	0.10%	4/8/2022 to 10/7/2022	7274	126,152
Lin, 2023^21^	USA	Outpatients 12+ years with COVID and at least 1 risk factor for severe infection	Tx: Mean 62 Cx: Mean 54	Tx: 60% Cx: 63%	NR	NR	0.66%	4/1/2022 to 2/20/2023	22,594	39,730
Low, 2023^61^	Malaysia	Outpatients diagnosed with COVID with at least one risk factor for severe disease	Mean: 48	53%	NR	0.64%	NR	7/14/2022 to 11/14/2022	10,483	10,483
Lui, 2023^29^	Hong Kong	Outpatients with type 2 diabetes and diagnosed with COVID-19	Mean: 72	50%	67.47%	67.5%	3.03%	2/26/2022 to 10/23/2022	793	793
Ma, 2023^32^	Hong Kong	Outpatients living in a nursing home and at high risk for severe disease with COVID-19	Mean: 83	60%	NR	59.6%	NR	2/16/2022 to 3/31/2022	483	8939
Paraskevis, 2023^62^	Greece	Outpatients age 65 and older diagnosed with COVID-19	75+: 59%	52%	NR	6.3%	1.87%	3/26/2022 to 7/20/2022	13,462	12,728
Park, 2022^31^	Republic of Korea	Outpatients in long term care facilities diagnosed with COVID-19	75+: 73%	52%	NR	NR	5.61%	2/6/2022 to 4/2/2022	623	196
Rajme-López, 2024^63^	Mexico	Outpatients diagnosed with COVID-19 with at least one risk factor for severe disease	Median: 60	63%	AC: 17.4% C19: 9.7%	NR	NR	1/1/2022 to 7/31/2022	332	783
Rodriguez-Leal, 2025^64^	Spain	Mild to moderate COVID-19 within 7 days of symptom onset presenting to ED	NR	51%	10.53%	NR	NR	1/1/2022 to 8/31/2022	224	1851
Sharif-Askari, 2024^65^	Dubai	Outpatients with mild to moderate COVID-19 and oxygen saturation ≥ 94%	Tx: median 54 Cx: median 37	54%	NR	2.4%	NR	5/22/2022 to 4/30/23	672	6618
Schwartz, 2023^38^	Canada	Outpatients with a positive test for COVID-19	Tx: Mean 74 Cx: Mean 52	63%	3.70%	NR	NR	4/4/2022 to 8/31/2022	8876	168,669
Shah, 2023^66^	USA	Outpatients diagnosed with COVID and with at least one risk factor for severe disease	18–49: 44.1% 50–64: 29.4% 65+: 26.5%	62%	NR	0.75%	NR	4/1/2022 to 8/31/2022	198,927	500,921
Shiau, 2024^67^	19 countries	Outpatients 40 and older with COPD and COVID-19	Mean: 68	55%	7.08%	6.23%	1.06%	1/1/2022 to 10/1/2023	5475	7994
Tsai 2023^68^	19 countries	Outpatients ≥ 65 years diagnosed with COVID-19	Mean: 73	58%	2.80%	2.50%	0.30%	1/1/2022 to 12/31/2022	28,824	28,824
Van Heer, 2023^69^	Australia	Outpatients 70+ years with COVID-19 and at least 1 vaccination	Mean: 77	55%	NR	11.9%	3.40%	7/11/2022 to 10/31/2022	5250	13,721
Wai, 2023^15^	Hong Kong	Outpatients with COVID-19	Tx: 60+ 93% Cx: 60+ 93%	53%	NR	NR	0.20%	2/22/2022 to 3/31/2022	14,121	23,430
Wee, 2023^70^	Singapore	Outpatients 60 and older diagnosed with COVID-19	Tx: 44% 60 to 69, 56% 70+ Cx: 60% 60 to 69, 40% 70+	56%	NR	1.70%	NR	3/18/2022 to 12/31/2022	3959	139,379
Wei, 2025^39^	Hong Kong	Outpatients with COVID-19 and at high risk for progression	Tx: mean 67 Cx: mean 69	57%	NR	2.22%	0.11%	3/16/2022 to 1/29/2023	93,861	46,616
Wong, 2022^71^	Hong Kong	Outpatients diagnosed with COVID-19 at high risk for severe disease	> 60: 85%	54%	NR	5.83%	0.60%	2/26/2022 to 6/26/2022	5542	54,672
Wu, 2023^72^	19 countries	Obese outpatient adults diagnosed with COVID-19	Mean: 57	66%	NR	2.46%	0.11%	1/1/2022 to 6/30/2022	30,969	30,969
Xie, 2023^40^	USA	Outpatients with COVID-19 and at least one risk factor for severe disease	Mean: 65	12%	4.61%	NR	NR	1/3/2022 to 11/30/2022	31,524	224,764
Yan, 2024^73^	USA	Outpatients with COVID-19 and at least one risk factor for severe disease	Median: 66	13%	2.60%	2.30%	0.30%	4/1/2022 to 3/31/2023	24,205	76,939
Yip, 2024^74^	Hong Kong	Outpatients with COVID-19 at risk for severe disease	Mean: 71	54%	NR	4.50%	NR	2/16/2022 to 3/31/2022	4921	83,154

*IPCW*, inverse probability of censoring weights; *PSM*, propensity score matching; *LR*, logistic regression; *IPTW*, inverse probability treatment weighting; *Tx*, treatment group; *Cx*, control group; *Nirm/rit*, nirmatrelvir/ritonavir; *Hosp*, hospitalization

### Study Quality Assessment

Using the NOS, we considered 28 studies to be of good quality in all three domains (selection, comparability and outcome/exposure). A total of 18 studies were judged to be of fair quality for not including a truly representative sample of the target population of our review (e.g., only certain age ranges, only people with diabetes mellitus, a chronic respiratory condition or obesity). Only one study was judged of poor quality because outcome assessment and follow-up adequacy were unclearly described. The full quality assessment is shown in Appendix Table 4.

### Main Results

**Figure 2:**
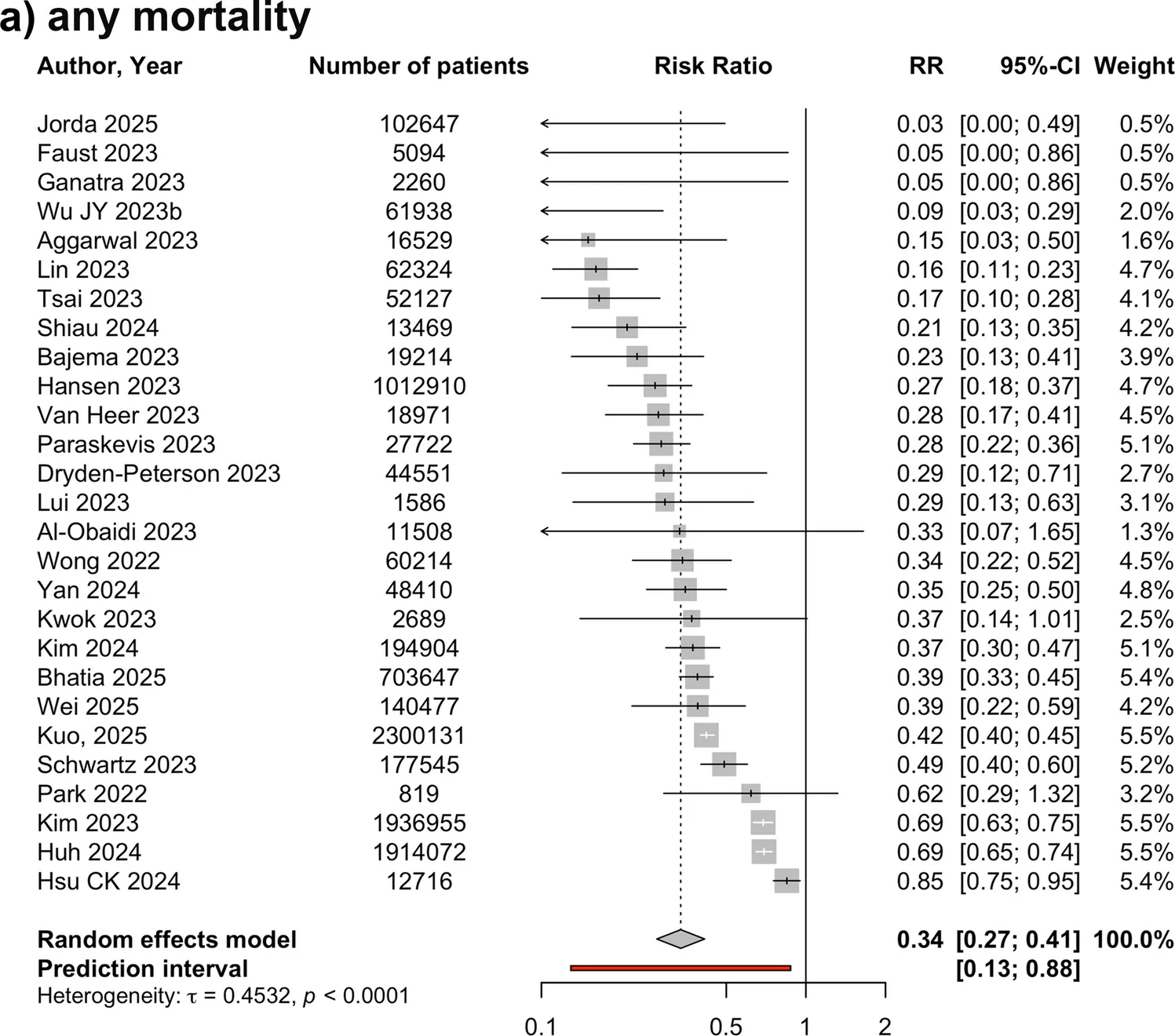

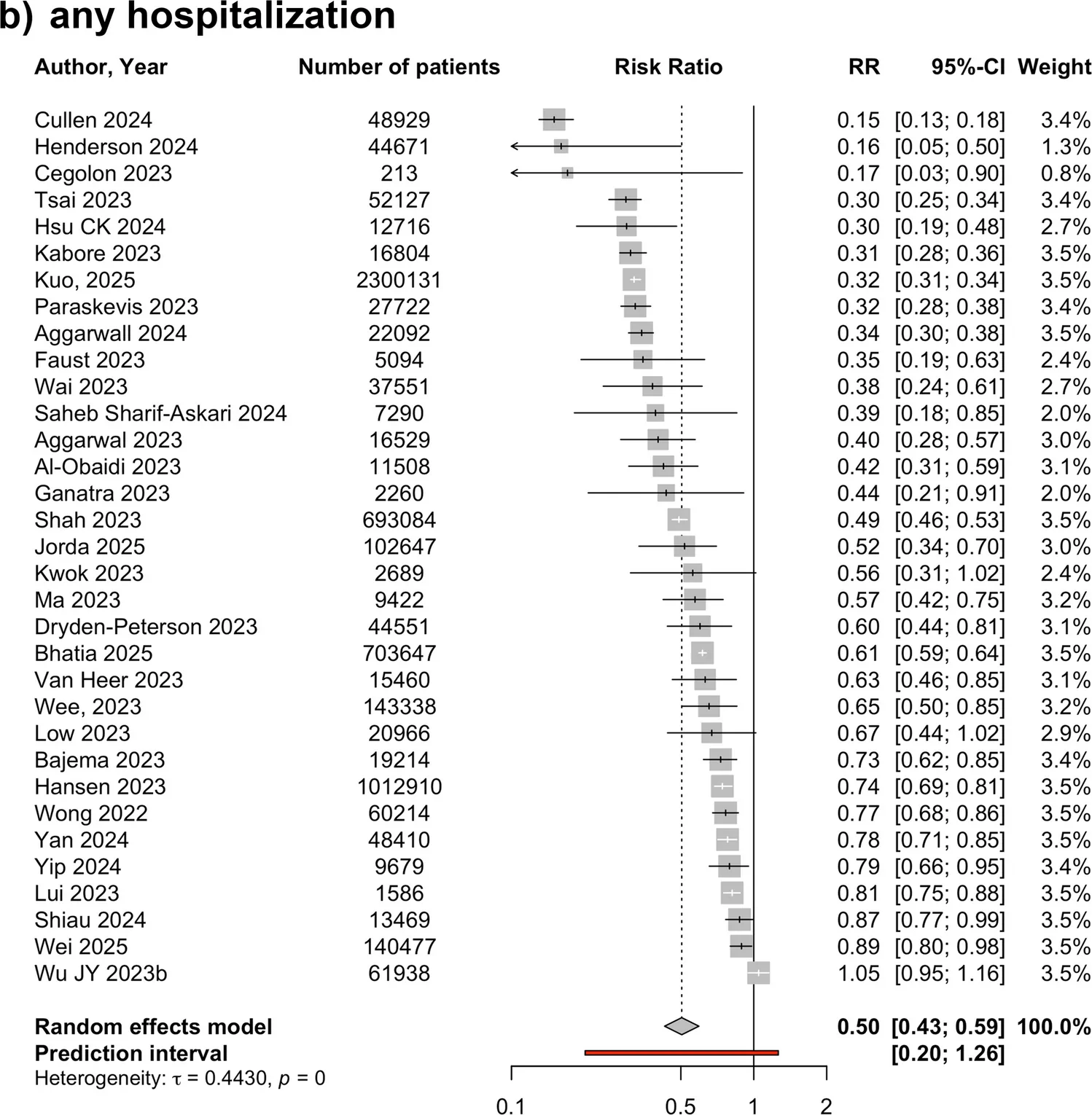

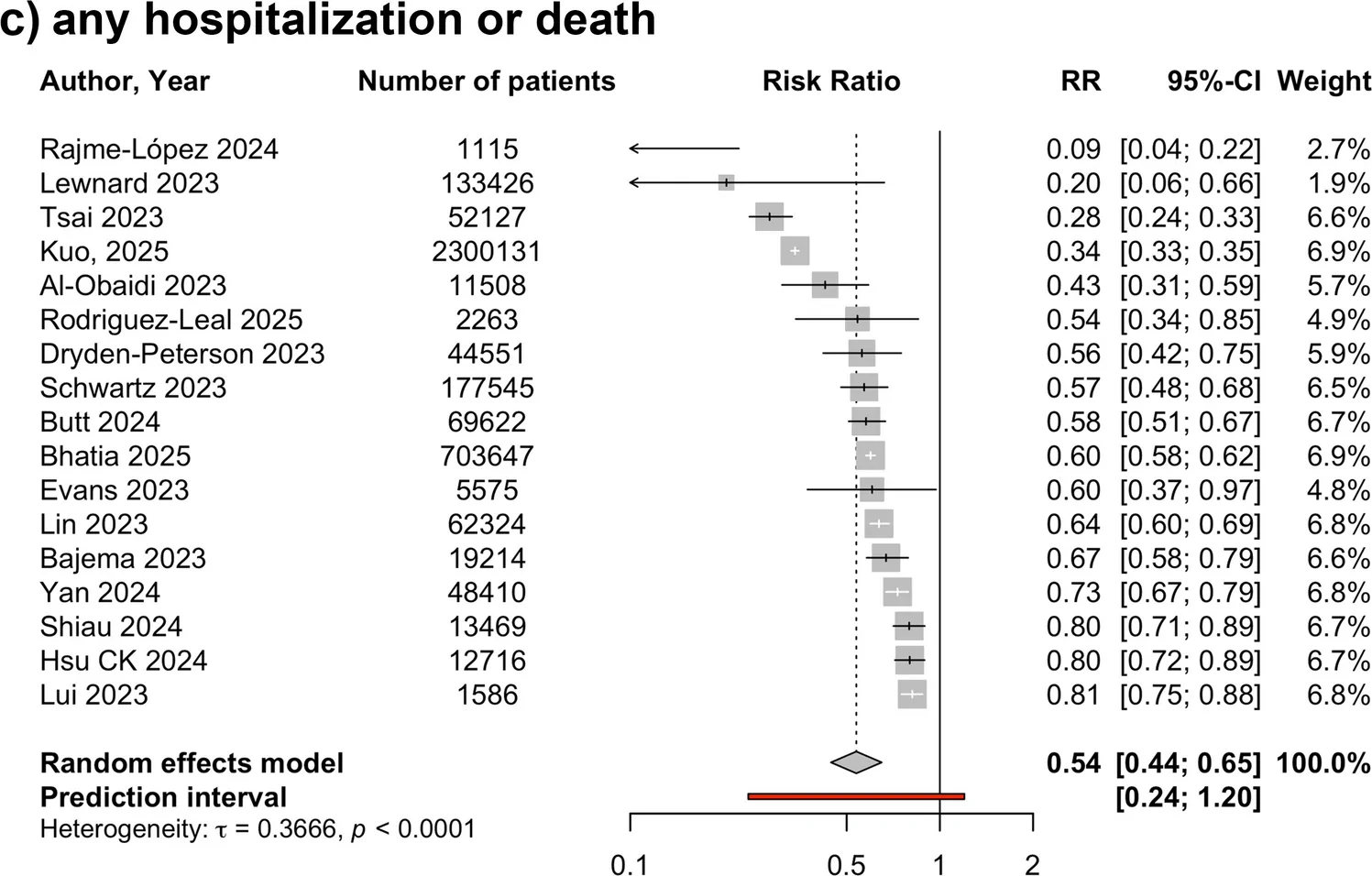
Forest plots for outcomes of (**a**) any mortality, (**b**) any hospitalization, and (**c**) the composite of any hospitalization or mortality.

The main results are summarized in Tables 2 and 3 and forest plots are shown in Fig. 2a–c. For patients treated with nirmatrelvir-ritonavir, pooled relative risks (RRs) were 0.54 (95% CI 0.43–0.68) for all-cause hospitalization, 0.45 (0.36–0.56) for COVID-19 hospitalization, and 0.50 (0.43–0.59) for any hospitalization. For mortality, pooled RRs were 0.30 (0.23–0.39) for 30-day all-cause mortality, 0.43 (0.32–0.59) for COVID-19 mortality, and 0.34 (0.27–0.41) for any mortality. The composite of hospitalization or death had an RR of 0.54 (0.44–0.65).

**Table 2:** Relative Risks of COVID-19 Versus All-Cause Clinical Outcomes for Patients Given Nirmatrelvir/Ritonavir Versus no Treatment. Boldface = Statistically Significant Finding

Outcome	No. of studies (No. of patients)	Relative risk (95% CI), *I*^2^	95% prediction interval; tau	*p *for comparison
All-cause hospitalization	20 (2,366,265)	**0.54 (0.43–0.68)**, 97%	0.19–1.54; 0.486	0.242
COVID-19 hospitalization	13 (3,343,073)	**0.45 (0.36–0.56)**, 96%	**0.21–0.96**; 0.331	
Any hospitalization outcome	33 (5,709,338)	**0.50 (0.43–0.59)**, 98%	0.20–1.26; 0.443	
All-cause mortality	20 (2,549,985)	**0.30 (0.23–0.39)**, 91%	**0.11–0.83**; 0.475	0.065
COVID-19 mortality	7 (6,395,444)	**0.43 (0.32–0.59)**, 97%	0.16–1.17; 0.377	
Any mortality outcome	27 (8,945,429)	**0.34 (0.27–0.41)**, 94%	**0.13–0.88;** 0.453	
Any composite of hospitalization or mortality	17 (3,659,229)	**0.54 (0.44–0.65)**, 99%	0.24–1.20; 0.367	

*CI*, confidence interval

**Table 3:** Estimated Absolute Risk Reduction and Number Needed to Treat to Prevent One Hospitalization for Low-, Moderate-, and High-Risk Outpatients

Risk group	Risk of hospitalization if untreated*	Risk of COVID-19 hospitalization with N-R^†^	Absolute risk reduction	Number needed to treat
Low	0.16%	0.07%	0.09%	1148
Moderate	2.2%	0.98%	1.2%	84
High	8.9%	4.0%	4.9%	20

^*^Based on Lehigh Outpatient COVID Hospitalization risk score during omicron phase of pandemic^3^.

^†^*N-R*, nirmatrelvir-ritonavir; assumes relative risk for COVID hospitalization of 0.45 based on analysis reported in Table 2

Corresponding 95% prediction intervals (PIs) were entirely below 1.0 for the outcomes of COVID-19 hospitalization (PI 0.21–0.96), all-cause mortality (0.11–0.83), and any mortality (0.13–0.88), indicating that for these outcomes the effects are likely to persist in subsequent studies.

Using risk strata from the LOCH score^3^ and applying the pooled RR for COVID-19 hospitalization (0.45, Table 2), the estimated absolute risk reductions were 0.09% (low risk), 1.2% (moderate risk), and 4.9% (high risk), corresponding to NNTs of 1148, 84, and 20, respectively (Table 3). This indicates that nirmatrelvir-ritonavir is likely to yield clinically meaningful absolute benefits primarily in patients at moderate or high baseline risk of complications.

### Subgroup Analyses

Prespecified subgroup analyses are summarized in Table 4 and in Appendix Figures 1 to 8. Because relative risks did not differ significantly for all-cause versus COVID-related hospitalization or all-cause versus COVID-related mortality (Table 2), subgroup analyses were conducted using the broader endpoints of any hospitalization and any mortality.

**Table 4:** Relative Risk of Experiencing Clinical Outcomes for Patients Given Nirmatrelvir/Ritonavir Versus No Treatment for Prespecified Subgroups. Boldface = Statistically Significant Finding

Subgroup	No. of studies (No. of patients)	Relative risk (95% CI)	Prediction interval; tau	*p* for comparison
**Any hospitalization**				
** Age group**				
18 to 39–49 years	3 (539,804)	**0.64 (0.57–0.72)**	**0.45–0.93**; 0.062	0.114
0–18 to 64 years	9 (2,059,241)	**0.55 (0.38–0.81)**	0.15–2.10; 0.543	
> 59–79 years	12 (1,521,193)	**0.47 (0.35–0.62)**	0.15–1.42; 0.484	
**Vaccination status**				
Not vaccinated	5 (418,187)	**0.36 (0.36–0.42)**	0.05–2.62; 0.647	0.5074
Primary series only	1 (149,498)	**0.50 (0.42–0.58)**	NA	
Primary series OR primary series + booster	4 (286,764)	**0.40 (0.27–0.59)**	0.11**–**1.42; 0.347	
Primary series + booster	3 (2,186,879)	**0.45 (0.37–0.54)**	**0.21–0.95**; 0.144	
Omicron phase*				
Before 1/1/2023	24 (3,658,093)	**0.46 (0.37–0.55)**	0.18–1.19; 0.451	**0.0049**
On or after 1/1/2023	9 (2,051,245)	**0.68 (0.56–0.82)**	0.35–1.29; 0.262	
**Immunocompromised**				
Yes	4 (81,556)	**0.53 (0.44–0.62)**	0.35–0.79; 0.094	0.468
No	2 (642,207)	**0.49 (0.45–0.53)**	**0.29–0.82**; 0.000	
**Risk level of population**^**†**^				
Low or average risk	20 (5,481,677)	**0.50 (0.42–0.60)**	0.22–1.15; 0.385	0.937
High risk	13 (227,661)	**0.51 (0.37–0.70)**	0.20–1.26; 0.443	
**PSM vs other method**^**‡**^				
PSM	19 (653,989)	**0.53 (0.42–0.68)**	0.18**–**1.60; 0.507	0.401
Other method	14 (6,055,349)	**0.47 (0.39–0.57)**	**0.23–0.95**; 0.311	
**Any mortality**				
** Age group**				
0–18 to 64 years	3 (1,684,687)	**0.23 (0.08–0.63)**	0.00–11.6; 0.750	0.491
> 59–79 years	7 (2,572,238)	**0.34 (0.23–0.50)**	0.09–1.30; 0.511	
**Vaccination status**				
Not vaccinated	4 (225,166)	**0.37 (0.29–0.46)**	**0.19–0.72**; 0.177	0.760
Primary series only	1 (170)	0.47 (0.17–1.29)	NA	
Primary series OR primary series + booster	3 (191,455)	**0.17 (0.03–0.82)**	0.00–51.0; 1.05	
Primary series + booster	4 (2,039,615)	**0.35 (0.18–0.70)**	0.03–3.76; 0.657	
Omicron phase*				
Before 1/1/2023	19 (6,653,925)	**0.34 (0.26–0.44)**	**0.13–0.91**; 0.446	0.880
On or after 1/1/2023	9 (2,291,504)	**0.33 (0.23–0.47)**	0.10–1.11; 0.493	
**Immunocompromised**				
Yes	1 (3627)	**0.26 (0.15–0.45)**	NA	**0.034**
No	1 (58,697)	**0.11 (0.06–0.19)**	NA	
**Risk level of population**^**†**^				
Low or average risk	16 (8,558,616)	**0.36 (0.27–0.49)**	0.12–1.11; 0.500	0.286
High risk	11 (386,813)	**0.29 (0.23–0.38)**	**0.13–0.65**; 0.331	
**PSM vs other method**^**‡**^				
PSM	17 (2,640,625)	**0.32 (0.23–0.45)**	0.10**–**1.08; 0.547	0.813
Other method	10 (6,304,804)	**0.34 (0.26–0.44)**	0.14**–**0.84; 0.379	
**Any hospitalization or mortality**				
**Age group**				
0–18 to 64 years	8 (1,762,110)	**0.65 (0.55–0.77)**	0.42–1.01; 0.162	0.120
18 to 39–49 years	1 (3867)	0.67 (0.39–1.18)	NA	
> 59–79 years	10 (994,900)	**0.52 (0.42–0.63)**	0.24–1.09; 0.315	
**Vaccination status**				
Not vaccinated	8 (249,296)	**0.50 (0.36–0.68)**	0.17–1.42; 0.415	0.128
Primary series only	2 (23,638)	**0.74 (0.59–0.93)**	0.11–4.95; 0.093	
Primary series OR primary series + booster	3 (169,603)	**0.65 (0.55–0.76)**	**0.46–0.92;** 0.001	
Primary series + booster	8 (2,269,345)	**0.56 (0.47–0.67)**	**0.32–0.99**; 0.221	
Omicron phase*				
Before 1/1/2023	11 (2,749,041)	**0.45 (0.33–0.60)**	0.16–1.27; 0.445	**0.0078**
On or after 1/1/2023	6 (910,188)	**0.68 (0.61–0.76)**	**0.47–0.99**; 0.132	
**Immunocompromised**				
Yes	7 (11,443)	**0.59 (0.51–0.69)**	**0.47–0.75**; 0.036	**0.021**
No	4 (108,913)	**0.64 (0.50–0.81)**	0.29–1.44; 0.222	
**Risk level of population**^**†**^				
Low or average risk	8 (3,306,516)	**0.48 (0.36–0.65)**	0.18–1.31; 0.394	0.129
High risk	9 (352,713)	**0.63 (0.54–0.73)**	0.38–1.04; 0.204	
**PSM vs other method**^**‡**^				
PSM	9 (337,690)	**0.53 (0.37–0.76)**	0.15**–**1.94; 0.531	0.936
Other method	8 (3,321,539)	**0.52 (0.43–0.63)**	**0.29–0.95**; 0.234	

^*^Date represents final day of data collection before or after this date

^†^Low-risk study populations had mortality < 0.5% in the untreated or control group. If mortality was not reported for a study a hospitalization rate < 4.0% in the untreated group was used

^‡^*PSM*, propensity score matching; other methods to adjust analysis included logistic regression, Cox regression, and inverse probability treatment weighting

For hospitalization, the RR was significantly lower earlier in the Omicron era (RR 0.46 early vs 0.68 late, *p* = 0.0049), and a similar pattern was observed for the composite of hospitalization or mortality (RR 0.45 vs 0.68, *p* = 0.0078).

For mortality, the RR was significantly lower for non-immunocompromised than for immunocompromised patients (RR 0.11 vs 0.26, *p* = 0.034), as so for the composite of hospitalization or mortality (RR 0.59 vs 0.64, *p* = 0.021). No other subgroup comparisons showed significant differences for any of the outcomes.

### Sensitivity Analysis

The sensitivity analysis comparing studies judged to be of good quality with those judged to be fair or poor quality found that they had a slightly higher RR for any mortality (0.39 vs 0.27, *p* = 0.031) but no significant differences were observed for any hospitalization (0.46 vs 0.57, *p* = 0.18) or the composite of any hospitalization or mortality (0.56 vs 0.49, *p* = 0.61).

Ten studies explicitly addressed immortal time bias in the analysis^29,30,33,35–40^. There was no difference in relative risk between studies that did and did not address immortal time bias for outcomes of hospitalization (0.57 vs 0.50, *p* = 0.68), mortality (0.34 vs 0.32, *p* = 0.35), or the composite of hospitalization or mortality (0.65 vs 0.48, *p* = 0.07).

### Evaluation for Publication Bias

Funnel plots are shown in Appendix Figure 9 for the outcomes of mortality, hospitalization and the composite of hospitalization and mortality. Larger studies were roughly symmetrically arranged around the summary estimates of relative risk. However, there were too few small studies to detect publication bias due to small study effects.

## DISCUSSION

This updated analysis of 47 cohort studies, all adjusted for important covariates, found that nirmatrelvir-ritonavir (N-R) is likely associated with a reduction in the likelihood of any mortality (RR 0.34, 95% prediction interval [PI] 0.13–0.88), all-cause mortality (RR 0.30, 95% PI 0.11–0.83) and in COVID-specific hospitalization (RR 0.45, 95% PI 0.21–0.96). Prediction intervals capture both within and between-study heterogeneity and are a more conservative way to summarize the effect of an intervention than confidence intervals. In addition, confidence intervals for almost all relative risks in the main results and subgroups were less than 1.0. Thus, there is still a substantial relative reduction in important mortality and hospitalization outcomes in the era of omicron variants and high levels of previous vaccination and infection.

One randomized trial was set at least in part during the omicron phase, with 1296 patients recruited between 8/25/2021 and 7/25/2022, overlapping with the late delta and early omicron waves of COVID-19^2^. Hospitalization was numerically lower in the N-R group (0.8% vs 1.6%, RR 0.5, 95% CI 0.17–1.46), but with the small number of events this analysis was underpowered. Nevertheless, the relative risk of 0.50 is consistent with the relative risk of 0.45 seen in our analysis for COVID-related hospitalization. A more recent randomized trial found no significant reduction in hospitalization with N-R, but the confidence intervals were broad and the study did find a significant reduction in duration of symptoms^75^.

In subgroup analyses, all point estimates for relative risk for all subgroups and outcomes were less than 0.75, and most had a 95% CI that excluded 1.0. The relative risk for any hospitalization was significantly higher later in the omicron phase than earlier (RR 0.46 for early versus 0.68 for late omicron, *p*= 0.005), consistent with less benefit. This may be due to increased immunity due to vaccination and previous infections, changing prescribing patterns, and changes in the virulence of the virus. This temporal trend highlights the need for ongoing study and surveillance of the effectiveness of N-R and other treatments. For example, while molnupiravir was initially shown to be effective in outpatients^41^, the PANORAMIC placebo-controlled randomized trial with 25,054 patients found no evidence of effectiveness in a more contemporary cohort (recruitment began 12/8/21), with 1% hospitalized in both the molnupiravir and placebo groups^42^.

While the relative risk was lower for non-immunocompromised than for immunocompromised patients (0.11 vs 0.26), this should be interpreted cautiously. These calculations were based on a relatively small number of studies, residual confounding is possible, and for both groups, the reduction in risk was clinically significant. Also, the definition of immunocompromised varied considerably between studies (Appendix Table 5).

The relative risk was consistent in studies with lower and higher levels of hospitalization and mortality in the control group (Appendix Fig. 6). However, the absolute benefit varies considerably based on the baseline level of risk. This baseline risk can be estimated in outpatients using the 3-item LOCH risk score that was prospectively validated in 19,456 outpatients during the omicron phase of the pandemic^3^. Table 3 summarizes the absolute benefit and number needed to treat to prevent hospitalization determined by applying the overall estimate of relative risk to different levels of baseline risk.

The *I*^2^ statistic is commonly used as a measure of between-study heterogeneity, and in our meta-analysis, the *I*^2^ values were largely over 90% indicating substantial heterogeneity (Table 3). However, this statistic has limited validity when very large studies with very narrow confidence intervals are included in a meta-analysis. In this case, calculation of the prediction interval that incorporates both within and between-study heterogeneity is recommended^23^. Visual inspection of the forest plots also shows that almost all relative risks and their 95% confidence intervals were less than 1.0 for all three outcomes.

### Strengths and Limitations

Strengths of the review include the large number of studies and patients, the consistent direction of effect, with nearly all included studies showing reduced hospitalization and mortality, and the geographic diversity of populations. To our knowledge, this is the most comprehensive and largest evidence synthesis to date and the only one to focus exclusively on the contemporary Omicron era, with widespread vaccination and prior infection. The finding that prediction intervals for mortality outcomes and COVID-related hospitalization lie entirely below 1.0 provides confidence that future studies are unlikely to significantly alter our conclusions.

Unmeasured confounding is always an issue with observational studies. We attempted to mitigate this by only using studies that reported an adjusted analysis, and the included studies generally adjusted for multiple covariates including age, multiple comorbidities, socioeconomic factors, and vaccination. Immortal time bias is a potential issue with retrospective cohort studies. But most studies reported that patients entered the study at the time that they were diagnosed with COVID-19, regardless of whether they were or were not treated. Also, there was no significant difference in relative risks between studies that did and did not explicitly address immortal time bias (although residual confounding can never be ruled out).

### Conclusions

We found a strong and consistent association between use of nirmatrelvir-ritonavir and a reduced likelihood of mortality and COVID-related hospitalization. The absolute reduction in risk of hospitalization is clinically meaningful in patients at moderate (NNT = 84) or high risk (NNT = 20) of complications.

## Supplementary Information

Below is the link to the electronic supplementary material.

ESM 1 (DOCX 5.21)

ESM 2 (DOCX 263 KB)

## Data Availability

The data used in the meta-analysis are available upon reasonable request.
