# Supplementary material for: Effectiveness of Nirmatrelvir/Ritonavir for Outpatients in the Era of Omicron, Vaccination, and Previous Infection: A Meta-analysis
Source: J Gen Intern Med. 2026 May 12;41(12):3516–30. doi: 10.1007/s11606-026-10494-4 (PMC13569784; doi:10.1007/s11606-026-10494-4)
Supplement: Supplementary file 1 [file 11606_2026_10494_MOESM1_ESM.docx]

**Appendices Table of Contents**

| **Name** | **Description** | **Page(s)** |
| --- | --- | --- |
| Appendix Text 1 | Search strategy | 2 |
| Appendix Table 1 | Description of how subgroups were created | 3-11 |
| Appendix Table 2 | Additional study characteristics | 12-15 |
| Appendix Table 3 | Adapted Newcastle – Ottawa assessment scale for cohort studies | 16 |
| Appendix Table 4 | Quality assessment of included studies according to NOS and AHRQ. | 17-19 |
| Appendix Table 5 | Definitions of immunocompromise used by individual studies | 20 |
| Appendix Figure 1 | Forest plots for all patients, all-cause and COVID-specific mortality | 21 |
| Appendix Figure 2 | Forest plots for all patients, all-cause and COVID-specific hospitalization | 21 |
| Appendix Figure 3 | Forest plots for any mortality, any hospitalization and composite of mortality or hospitalization stratified by age | 22 |
| Appendix Figure 4 | Forest plots for any mortality, any hospitalization and composite of mortality or hospitalization stratified by early versus late stage of omicron | 23 |
| Appendix Figure 5 | Forest plots for any mortality, any hospitalization and composite of mortality or hospitalization stratified by immunocompromise | 24 |
| Appendix Figure 6 | Forest plots for any mortality, any hospitalization and composite of mortality or hospitalization stratified by low vs high risk of outcomes in untreated group | 25 |
| Appendix Figure 7 | Forest plots for any mortality, any hospitalization and composite of mortality or hospitalization stratified by propensity score matching vs other adjustment method | 26 |
| Appendix Figure 8 | Forest plots for any mortality, any hospitalization and composite of mortality or hospitalization stratified by vaccination history | 27 |
| Appendix Figure 9 | Funnel plots for outcomes of mortality, hospitalization and composite of mortality and hospitalization | 30 |

**Appendix Text 1. Search strategy**

PubMed search for observational studies

("nirmaltrelvir" OR "nirmatrelvir-ritonavir" OR "nirmatrelvir/ritonavir" OR "Paxlovid" OR "paxlovid")

AND

(“randomized” OR “controlled trial” OR "propensity score" OR "propensity-score" OR "matched" OR "cohort" OR "case-control" OR "case control" OR "case cohort" OR "case-cohort" OR "observational")

AND

("hospitalization" OR "hospital" OR "hospitalized" OR "hospitalized" OR "hospitalization" OR "death" OR "mortality" OR "emergency department" OR "intensive care" OR "ICU").

Cochrane central search

Nirmatrelvir OR paxlovid

Appendix Table 1. Original and combined subgroups.

| **AuthorYear** | **PMID** | **Original subgroup** | **Combined subgroup name** |
| --- | --- | --- | --- |
| Aggarwal 2023 | 36780912 | Age18to64 | Age0_18to64 |
| Aggarwal 2023 | 36780912 | AgeOver64 | AgeOver64 |
| Aggarwal 2023 | 36780912 | All | All |
| Aggarwal 2023 | 36780912 | All | All |
| Aggarwal 2023 | 36780912 | All | All |
| Aggarwal 2023 | 36780912 | Comorbid0to1 | Comorbid0to1 |
| Aggarwal 2023 | 36780912 | ComorbidOver1 | ComorbidOver1 |
| Aggarwal 2023 | 36780912 | ImmunocompromisedMild | ImmunocompromisedMild |
| Aggarwal 2023 | 36780912 | ImmunocompromisedModToSevere | ImmunocompromisedModToSevere |
| Aggarwal 2023 | 36780912 | ImmunocompromisedNo | ImmunocompromisedNo |
| Aggarwal 2023 | 36780912 | ImmunocompromisedYes | ImmunocompromisedYes |
| Aggarwal 2023 | 36780912 | ObeseNo | ObeseNo |
| Aggarwal 2023 | 36780912 | ObeseYes | ObeseYes |
| Aggarwal 2023 | 36780912 | OmicronBeforeBA4andBA5 | OmicronBeforeBA4andBA5 |
| Aggarwal 2023 | 36780912 | OmicronDuringBA4andBA5 | OmicronDuringBA4andBA5 |
| Aggarwal 2023 | 36780912 | Vaccinated0 | VaccinatedNo |
| Aggarwal 2023 | 36780912 | Vaccinated0to2 | VaccinatedDisregard |
| Aggarwal 2023 | 36780912 | Vaccinated1to2 | VaccinatedDisregard |
| Aggarwal 2023 | 36780912 | VaccinatedOver2 | VaccinatedBoosted |
| Aggarwall 2024 | 38500962 | All | All |
| Al-Obaidi 2023 | 36898600 | Age18to64 | Age0_18to64 |
| Al-Obaidi 2023 | 36898600 | AgeOver64 | AgeOver64 |
| Al-Obaidi 2023 | 36898600 | All | All |
| Al-Obaidi 2023 | 36898600 | All | All |
| Al-Obaidi 2023 | 36898600 | All | All |
| Al-Obaidi 2023 | 36898600 | ImmunocompromisedNo | ImmunocompromisedNo |
| Al-Obaidi 2023 | 36898600 | ImmunocompromisedYes | ImmunocompromisedYes |
| Al-Obaidi 2023 | 36898600 | Vaccinated0to1 | VaccinatedNo |
| Al-Obaidi 2023 | 36898600 | VaccinatedOver2 | VaccinatedBoosted |
| Arbel 2022 | 36001529 | Age40to64 | Age0_18to64 |
| Arbel 2022 | 36001529 | Age40to64ImmunityNo | Age0to64ImmunityNo |
| Arbel 2022 | 36001529 | Age40to64ImmunityYes | Age0to64ImmunityYes |
| Arbel 2022 | 36001529 | AgeOver64 | AgeOver64 |
| Arbel 2022 | 36001529 | AgeOver64ImmunityNo | AgeOver64ImmunityNo |
| Arbel 2022 | 36001529 | AgeOver64ImmunityYes | AgeOver64ImmunityYes |
| Bajema 2023 | 37276589 | Age18to64 | Age0_18to64 |
| Bajema 2023 | 37276589 | AgeOver64 | AgeOver64 |
| Bajema 2023 | 37276589 | All | All |
| Bajema 2023 | 37276589 | All | All |
| Bajema 2023 | 37276589 | All | All |
| Bajema 2023 | 37276589 | ImmunocompromisedNo | ImmunocompromisedNo |
| Bajema 2023 | 37276589 | ImmunocompromisedYes | ImmunocompromisedYes |
| Bajema 2023 | 37276589 | SymptomsNo | SymptomsNo |
| Bajema 2023 | 37276589 | SymptomsYes | SymptomsYes |
| Bajema 2023 | 37276589 | Tx0to1days | Tx0to1days |
| Bajema 2023 | 37276589 | Tx2to5days | Tx2to5days |
| Bajema 2023 | 37276589 | VaccinatedNo | VaccinatedNo |
| Bajema 2023 | 37276589 | VaccinatedYes | VaccinatedPrimaryOrBooster |
| Bhatia 2025 | 39823513 | Age18to49 | Age18to39_49 |
| Bhatia 2025 | 39823513 | Age50to64 | Age0_18to64 |
| Bhatia 2025 | 39823513 | AgeOver64 | AgeOver64 |
| Bhatia 2025 | 39823513 | All | All |
| Bhatia 2025 | 39823513 | All | All |
| Bhatia 2025 | 39823513 | All | All |
| Bhatia 2025 | 39823513 | Vaccinated0 | VaccinatedNo |
| Bhatia 2025 | 39823513 | VaccinatedOver1 | VaccinatedPrimaryOrBooster |
| Butt 2024 | 37711076 | Age18to60 | Age0_18to64 |
| Butt 2024 | 37711076 | AgeOver60 | AgeOver64 |
| Butt 2024 | 37711076 | All | All |
| Butt 2024 | 37711076 | Black | Black |
| Butt 2024 | 37711076 | Cancer | Cancer |
| Butt 2024 | 37711076 | CardiovascularYes | CardiovascularYes |
| Butt 2024 | 37711076 | ChronicKidneyDisease | ChronicKidneyDisease |
| Butt 2024 | 37711076 | ChronicLungDisease | ChronicLungDisease |
| Butt 2024 | 37711076 | DiabetesYes | DiabetesYes |
| Butt 2024 | 37711076 | Female | Female |
| Butt 2024 | 37711076 | Male | Male |
| Butt 2024 | 37711076 | ObeseYes | ObeseYes |
| Butt 2024 | 37711076 | OtherUnknownRace | OtherUnknownRace |
| Butt 2024 | 37711076 | SymptomsNo | SymptomsNo |
| Butt 2024 | 37711076 | SymptomsYes | SymptomsYes |
| Butt 2024 | 37711076 | Vaccinated0to1 | VaccinatedNo |
| Butt 2024 | 37711076 | Vaccinated2 | VaccinatedPrimary |
| Butt 2024 | 37711076 | Vaccinated3 | VaccinatedBoosted |
| Butt 2024 | 37711076 | White | White |
| Cegolon 2023 | 37242504 | All | All |
| Cullen 2024 | 39649410 | Age12to64 | Age0_18to64 |
| Cullen 2024 | 39649410 | AgeOver64 | AgeOver64 |
| Cullen 2024 | 39649410 | All | All |
| Cullen 2024 | 39649410 | VaccinatedOver0 | VaccinatedDisregard |
| Dormuth 2023 | 37782496 | HighRisk | HighRisk |
| Dormuth 2023 | 37782496 | ImmunocompromisedMod | ImmunocompromisedMod |
| Dormuth 2023 | 37782496 | ImmunocompromisedSevere | ImmunocompromisedSevere |
| Dormuth 2023 | 37782496 | ModerateRisk | ModerateRisk |
| Dryden-Peterson 2023 | 36508742 | Age50to64 | Age0_18to64 |
| Dryden-Peterson 2023 | 36508742 | AgeOver64 | AgeOver64 |
| Dryden-Peterson 2023 | 36508742 | All | All |
| Dryden-Peterson 2023 | 36508742 | All | All |
| Dryden-Peterson 2023 | 36508742 | All | All |
| Dryden-Peterson 2023 | 36508742 | ObeseNo | ObeseNo |
| Dryden-Peterson 2023 | 36508742 | ObeseYes | ObeseYes |
| Dryden-Peterson 2023 | 36508742 | Vaccinated0to1 | VaccinatedNo |
| Dryden-Peterson 2023 | 36508742 | VaccinatedOver1 | VaccinatedPrimaryOrBooster |
| Evans 2023 | 36773891 | All | All |
| Faust 2023 | 37387690 | All | All |
| Faust 2023 | 37387690 | All | All |
| Faust 2023 | 37387690 | VaccinatedYes | VaccinatedYes |
| Faust 2023 | 37387690 | VaccinatedYes | VaccinatedYes |
| Ganatra 2023 | 35986628 | All | All |
| Ganatra 2023 | 35986628 | All | All |
| Ganatra 2023 | 35986628 | VaccinatedYes | VaccinatedYes |
| Ganatra 2023 | 35986628 | VaccinatedYes | VaccinatedYes |
| Hammond 2022 | 35172054 | Age18to64 | Age0_18to64 |
| Hammond 2022 | 35172054 | AgeOver64 | AgeOver64 |
| Hammond 2022 | 35172054 | All | All |
| Hammond 2022 | 35172054 | All | All |
| Hammond 2022 | 35172054 | All | All |
| Hammond 2022 | 35172054 | Female | Female |
| Hammond 2022 | 35172054 | Male | Male |
| Hammond 2022 | 35172054 | ObeseNo | ObeseNo |
| Hammond 2022 | 35172054 | ObeseYes | ObeseYes |
| Hammond 2024 | 38598573 | All | All |
| Hammond 2024 | 38598573 | All | All |
| Hammond 2024 | 38598573 | All | All |
| Hansen 2023 | 37398261 | All | All |
| Hansen 2023 | 37398261 | All | All |
| Henderson 2024 | 38380946 | All | All |
| Hsu CK 2024 | 38568820 | All | All |
| Hsu CK 2024 | 38568820 | All | All |
| Hsu CK 2024 | 38568820 | All | All |
| Huh 2024 | 39497563 | All | All |
| Jorda 2025 | 39505067 | All | All |
| Jorda 2025 | 39505067 | All | All |
| Kabore 2023 | 37149726 | Age0to69 | Age0_18to64 |
| Kabore 2023 | 37149726 | AgeOver69 | AgeOver64 |
| Kabore 2023 | 37149726 | All | All |
| Kabore 2023 | 37149726 | ImmunocompromisedYes | ImmunocompromisedYes |
| Kabore 2023 | 37149726 | Vaccinated0to1 | VaccinatedNo |
| Kabore 2023 | 37149726 | VaccinatedOver1 | VaccinatedPrimaryOrBooster |
| Kim 2023 | 37431541 | AgeOver59 | AgeOver64 |
| Kim 2023 | 37431541 | AgeOver69 | AgeOver64 |
| Kim 2023 | 37431541 | AgeOver79 | AgeOver64 |
| Kim 2023 | 37431541 | All | All |
| Kim 2023 | 37431541 | Vaccinated0 | VaccinatedNo |
| Kim 2023 | 37431541 | VaccinatedOver0 | VaccinatedDisregard |
| Kim 2024 | 38621766 | AgeOver59 | AgeOver64 |
| Kim 2024 | 38621766 | AgeOver69 | AgeOver64 |
| Kim 2024 | 38621766 | AgeOver79 | AgeOver64 |
| Kim 2024 | 38621766 | All | All |
| Kim 2024 | 38621766 | Vaccinated0 | VaccinatedNo |
| Kim 2024 | 38621766 | VaccinatedOver0 | VaccinatedPrimaryOrBooster |
| Kuo, 2025 | 40988922 | Age18to64 | Age0_18to64 |
| Kuo, 2025 | 40988922 | Age18to64 | Age0_18to64 |
| Kuo, 2025 | 40988922 | Age18to64 | Age0_18to64 |
| Kuo, 2025 | 40988922 | AgeOver64 | AgeOver64 |
| Kuo, 2025 | 40988922 | AgeOver64 | AgeOver64 |
| Kuo, 2025 | 40988922 | AgeOver64 | AgeOver64 |
| Kuo, 2025 | 40988922 | All | All |
| Kuo, 2025 | 40988922 | All | All |
| Kuo, 2025 | 40988922 | All | All |
| Kuo, 2025 | 40988922 | Vaccinated1to2 | VaccinatedDisregard |
| Kuo, 2025 | 40988922 | Vaccinated1to2 | VaccinatedDisregard |
| Kuo, 2025 | 40988922 | Vaccinated1to2 | VaccinatedDisregard |
| Kuo, 2025 | 40988922 | Vaccinated3up | VaccinatedBoosted |
| Kuo, 2025 | 40988922 | Vaccinated3up | VaccinatedBoosted |
| Kuo, 2025 | 40988922 | Vaccinated3up | VaccinatedBoosted |
| Kuo, 2025 | 40988922 | VaccinatedNo | VaccinatedNo |
| Kuo, 2025 | 40988922 | VaccinatedNo | VaccinatedNo |
| Kuo, 2025 | 40988922 | VaccinatedNo | VaccinatedNo |
| Kwok 2023 | 36992319 | All | All |
| Kwok 2023 | 36992319 | All | All |
| Lewnard 2023 | 36933565 | All | All |
| Lewnard 2023 | 36933565 | VaccinatedOver1 | VaccinatedPrimaryOrBooster |
| Lewnard, 2023 | 36933565 | VaccinatedOver2 | VaccinatedBoosted |
| Lin 2023 | 37733342 | Age12To64 | Age0_18to64 |
| Lin 2023 | 37733342 | Age12To64 | Age0_18to64 |
| Lin 2023 | 37733342 | AgeOver64 | AgeOver64 |
| Lin 2023 | 37733342 | AgeOver64 | AgeOver64 |
| Lin 2023 | 37733342 | All | All |
| Lin 2023 | 37733342 | All | All |
| Lin 2023 | 37733342 | CardiovascularNo | CardiovascularNo |
| Lin 2023 | 37733342 | CardiovascularNo | CardiovascularNo |
| Lin 2023 | 37733342 | CardiovascularYes | CardiovascularYes |
| Lin 2023 | 37733342 | CardiovascularYes | CardiovascularYes |
| Lin 2023 | 37733342 | DiabetesNo | DiabetesNo |
| Lin 2023 | 37733342 | DiabetesNo | DiabetesNo |
| Lin 2023 | 37733342 | DiabetesYes | DiabetesYes |
| Lin 2023 | 37733342 | DiabetesYes | DiabetesYes |
| Lin 2023 | 37733342 | ImmunocompromisedNo | ImmunocompromisedNo |
| Lin 2023 | 37733342 | ImmunocompromisedNo | ImmunocompromisedNo |
| Lin 2023 | 37733342 | ImmunocompromisedYes | ImmunocompromisedYes |
| Lin 2023 | 37733342 | ImmunocompromisedYes | ImmunocompromisedYes |
| Lin 2023 | 37733342 | NonWhite | NonWhite |
| Lin 2023 | 37733342 | NonWhite | NonWhite |
| Lin 2023 | 37733342 | ObeseNo | ObeseNo |
| Lin 2023 | 37733342 | ObeseNo | ObeseNo |
| Lin 2023 | 37733342 | ObeseYes | ObeseYes |
| Lin 2023 | 37733342 | ObeseYes | ObeseYes |
| Lin 2023 | 37733342 | Vaccinated0to2 | VaccinatedDisregard |
| Lin 2023 | 37733342 | Vaccinated0to2 | VaccinatedDisregard |
| Lin 2023 | 37733342 | VaccinatedOver2 | VaccinatedBoosted |
| Lin 2023 | 37733342 | VaccinatedOver2 | VaccinatedBoosted |
| Lin 2023 | 37733342 | White | White |
| Lin 2023 | 37733342 | White | White |
| Low 2023 | 37567557 | Age18to39 | Age18to39_49 |
| Low 2023 | 37567557 | Age40to59 | Age0_18to64 |
| Low 2023 | 37567557 | AgeOver59 | AgeOver64 |
| Low 2023 | 37567557 | All | All |
| Low 2023 | 37567557 | Female | Female |
| Low 2023 | 37567557 | Male | Male |
| Lui 2023 | 37204790 | All | All |
| Lui 2023 | 37204790 | All | All |
| Lui 2023 | 37204790 | All | All |
| Ma 2023 | 37103932 | All | All |
| Paraskevis 2023 | 37565522 | All | All |
| Paraskevis 2023 | 37565522 | All | All |
| Park 2022 | 36617550 | All | All |
| Park 2022 | 36617550 | Vaccinated2 | VaccinatedPrimary |
| Park 2022 | 36617550 | Vaccinated3 | VaccinatedBoosted |
| Rajme-López 2024 | 38545451 | All | All |
| Rodriguez-Leal 2025 | 40412101 | All | All |
| Rodriguez-Leal 2025 | 40412101 | ImmunocompromisedYes | ImmunocompromisedYes |
| Saheb Sharif-Askari 2024 | 39472619 | All | All |
| Schilling 2024 | 37778363 | All | All |
| Schwartz 2023 | 36781188 | Age18to69 | Age0_18to64 |
| Schwartz 2023 | 36781188 | Age18to69 | Age0_18to64 |
| Schwartz 2023 | 36781188 | AgeOver69 | AgeOver64 |
| Schwartz 2023 | 36781188 | AgeOver69 | AgeOver64 |
| Schwartz 2023 | 36781188 | All | All |
| Schwartz 2023 | 36781188 | All | All |
| Schwartz 2023 | 36781188 | HighRisk | HighRisk |
| Schwartz 2023 | 36781188 | HighRisk | HighRisk |
| Schwartz 2023 | 36781188 | StandardRisk | StandardRisk |
| Schwartz 2023 | 36781188 | StandardRisk | StandardRisk |
| Schwartz 2023 | 36781188 | Vaccinated0 | VaccinatedNo |
| Schwartz 2023 | 36781188 | Vaccinated0 | VaccinatedNo |
| Schwartz 2023 | 36781188 | Vaccinated1to2 | VaccinatedDisregard |
| Schwartz 2023 | 36781188 | Vaccinated1to2 | VaccinatedDisregard |
| Schwartz 2023 | 36781188 | VaccinatedOver2 | VaccinatedBoosted |
| Schwartz 2023 | 36781188 | VaccinatedOver2 | VaccinatedBoosted |
| Shah 2023 | 36454693 | Age18to49 | Age18to39_49 |
| Shah 2023 | 36454693 | Age18to49Vax0 | Age18to49Vax0 |
| Shah 2023 | 36454693 | Age18to49Vax2 | Age18to49Vax2 |
| Shah 2023 | 36454693 | Age18to49VaxOver2 | Age18to49VaxOver2 |
| Shah 2023 | 36454693 | Age50to64 | Age0_18to64 |
| Shah 2023 | 36454693 | Age50to64Vax0 | Age0to64Vax0 |
| Shah 2023 | 36454693 | Age50to64Vax2 | Age0to64Vax2 |
| Shah 2023 | 36454693 | Age50to64VaxOver2 | Age0to64VaxOver2 |
| Shah 2023 | 36454693 | AgeOver64 | AgeOver64 |
| Shah 2023 | 36454693 | AgeOver64Vax0 | AgeOver64Vax0 |
| Shah 2023 | 36454693 | AgeOver64Vax2 | AgeOver64Vax2 |
| Shah 2023 | 36454693 | AgeOver64VaxOver2 | AgeOver64VaxOver2 |
| Shah 2023 | 36454693 | All | All |
| Shah 2023 | 36454693 | ImmunocompromisedNo | ImmunocompromisedNo |
| Shah 2023 | 36454693 | ImmunocompromisedYes | ImmunocompromisedYes |
| Shah 2023 | 36454693 | Vaccinated0 | VaccinatedNo |
| Shah 2023 | 36454693 | Vaccinated2 | VaccinatedPrimary |
| Shah 2023 | 36454693 | VaccinatedOver2 | VaccinatedBoosted |
| Shiau 2024 | 38702925 | All | All |
| Shiau 2024 | 38702925 | All | All |
| Shiau 2024 | 38702925 | All | All |
| Tsai 2023 | 37322616 | AgeOver64 | AgeOver64 |
| Tsai 2023 | 37322616 | AgeOver64 | AgeOver64 |
| Tsai 2023 | 37322616 | AgeOver64 | AgeOver64 |
| Tsai 2023 | 37322616 | All | All |
| Tsai 2023 | 37322616 | All | All |
| Tsai 2023 | 37322616 | All | All |
| Van Heer 2023 | 37927380 | AgeOver69 | AgeOver64 |
| Van Heer 2023 | 37927380 | AgeOver69 | AgeOver64 |
| Van Heer 2023 | 37927380 | All | All |
| Van Heer 2023 | 37927380 | All | All |
| Wai 2023 | 36212676 | All | All |
| Wee 2023 | 37331509 | AgeOver59 | AgeOver64 |
| Wee, 2023 | 37331509 | All | All |
| Wee, 2023 | 37331509 | ImmunocompromisedYes | ImmunocompromisedYes |
| Wei 2025 | 39964106 | All | All |
| Wei 2025 | 39964106 | All | All |
| Wong 2022 | 36216007 | All | All |
| Wong 2022 | 36216007 | All | All |
| Wu JY 2023b | 37769748 | Age18to40 | Age18to39_49 |
| Wu JY 2023b | 37769748 | Age41to64 | Age0_18to64 |
| Wu JY 2023b | 37769748 | Age41to64 | Age0_18to64 |
| Wu JY 2023b | 37769748 | AgeOver64 | AgeOver64 |
| Wu JY 2023b | 37769748 | AgeOver64 | AgeOver64 |
| Wu JY 2023b | 37769748 | All | All |
| Wu JY 2023b | 37769748 | All | All |
| Wu JY 2023b | 37769748 | Female | Female |
| Wu JY 2023b | 37769748 | Male | Male |
| Wu JY 2023b | 37769748 | Male | Male |
| Wu JY 2023b | 37769748 | VaccinatedNo | VaccinatedNo |
| Wu JY 2023b | 37769748 | VaccinatedNo | VaccinatedNo |
| Wu JY 2023b | 37769748 | VaccinatedYes | VaccinatedYes |
| Xie 2023 | 37041016 | Age18to65 | Age0_18to64 |
| Xie 2023 | 37041016 | AgeOver65 | AgeOver64 |
| Xie 2023 | 37041016 | Black | Black |
| Xie 2023 | 37041016 | Female | Female |
| Xie 2023 | 37041016 | Male | Male |
| Xie 2023 | 37041016 | NoPrevInfection | NoPrevInfection |
| Xie 2023 | 37041016 | PrevInfection | PrevInfection |
| Xie 2023 | 37041016 | Vaccinated0 | VaccinatedNo |
| Xie 2023 | 37041016 | Vaccinated1to2 | VaccinatedDisregard |
| Xie 2023 | 37041016 | VaccinatedOver2 | VaccinatedBoosted |
| Xie 2023 | 37041016 | White | White |
| Yan 2024 | 38864601 | Age18to49 | Age18to39_49 |
| Yan 2024 | 38864601 | Age50to64 | Age0_18to64 |
| Yan 2024 | 38864601 | Age65to74 | AgeOver64 |
| Yan 2024 | 38864601 | AgeOver74 | AgeOver64 |
| Yan 2024 | 38864601 | All | All |
| Yan 2024 | 38864601 | All | All |
| Yan 2024 | 38864601 | All | All |
| Yan 2024 | 38864601 | ImmunocompromisedNo | ImmunocompromisedNo |
| Yan 2024 | 38864601 | ImmunocompromisedYes | ImmunocompromisedYes |
| Yan 2024 | 38864601 | Risk1of4 | Risk1of4 |
| Yan 2024 | 38864601 | Risk2of4 | Risk2of4 |
| Yan 2024 | 38864601 | Risk3of4 | Risk3of4 |
| Yan 2024 | 38864601 | Risk4of4 | Risk4of4 |
| Yan 2024 | 38864601 | Vaccinated2 | VaccinatedPrimary |
| Yan 2024 | 38864601 | VaccinatedNo | VaccinatedNo |
| Yan 2024 | 38864601 | VaccinatedOver2 | VaccinatedBoosted |
| Yip 2024 | 36031408 | Age18to69 | Age0_18to64 |
| Yip 2024 | 36031408 | AgeOver69 | AgeOver64 |
| Yip 2024 | 36031408 | All | All |
| Yip 2024 | 36031408 | Highrisk | HighRisk |

Appendix Table 2. Additional study characteristics

| **Author, year** | **Adjustment method** | **Variables adjusted for** | **Outcomes reported** | **Vaccination** |
| --- | --- | --- | --- | --- |
| Aggarwal, 2023 | PSM | Age, sex, insurance, obesity, immunocompromise, number of comorbidities, number of vaccinations, and omicron subvariant. | Hospitalization Death | 2 doses: 15% treated, 16% controls 3+ doses: 61% treated, 58% controls |
| Aggarwal, 2024 | LR and IPTW | Age, sex, race/ethnicity, comorbidities, immunocompromise, obesity, insurance, vaccination. | Hospitalization | None: 18% 1 dose: 4% 2 doses: 14.4% 3+ doses: 64% |
| Al-Obaidi, 2023 | PSM | Age, BMI, vaccination, diabetes, comorbidities. | Hospitalization Death Hospitalization or death | 3+ doses: 42% |
| Arbel, 2022 | Cox | Age, sex and comorbidities. | Hospitalization | Vaccinated (2+) or previous infection: 78% |
| Bajema, 2023 | PSM | Age, sex, race, region, vaccination, symptom number, substance use, comorbidities, health encounters. | Hospitalization Death Hospitalization or death | None: 17% 1 dose: 4% 2 doses: 28% 3+ doses: 51% |
| Bhatia, 2025 | IPCW | Age, sex, race/ethnicity, comorbidities, risk factors for severe COVID, hospitalizations in previous year. | Hospitalization Death Hospitalization or death | NR |
| Butt, 2024 | IPTW | Age, sex, race, BMI, vaccination, and comorbidities | Hospitalization or death | None or 1: 25% 2 doses: 26% 3+ doses: 49% |
| Cegelon, 2023 | LR | Age, sex, vaccination, Charlson index and immunosuppression. |  | 0 doses: 18% 1-2 doses: 17% 3+ doses: 65% |
| Cullen, 2024 | PSM | Age, sex, race, ethnicity, region, insurance type, cohort entry month, healthcare utilization, COVID-19 vaccination status, number of past SARS-CoV-2 infections, and comorbidities. | Hospitalization | Any: 67% |
| Dormuth, 2023 | PSM | Age, sex, and propensity score. | Hospitalization or death | Group 1: 90% 3 or more Group 2: 80% 3 or more Group 3: 83% 3 or more Group 4: 62% 3 or more |
| Dryden-Peterson 2023 | IPTW | Age, vaccination, comorbidities, neighorhood depreivation, and race/ethnicity. | Hospitalization Death Hospitalization or death | 0: 8% 1: 2% 2: 25% 3+: 66% |
| Evans 2023 | Coxl | Age, sex, comorbidities, vaccination and social deprivation. | Hospitalization or death | Treated: 2% 0, 62% 1 to 3, 36% 4+ Control: 4% 0, 78% 1 to 3, 18% 4+ |
| Faust, 2023 | PSM | Age, sex, race, BMI, comorbidities, medications, lab values | Hospitalization Death | 100% vaccinated |
| Ganatra, 2023 | PSM | Age, sex, race, BMI, comorbidities, medications, lab values | Hospitalization Death | NR |
| Hammond, 2022 | NA | NA | Hospitalization Death Hospitalization or death |  |
| Hammond, 2024 | NA | NA | Hospitalization Death Hospitalization or death | Vaccinated (2+): 57% |
| Hansen, 2023 | IPTW | Age, sex, race, BMI, comorbidities, risk factors for severe COVID, and deprivation by zip code. | Hospitalization Death | Treated: 40% 0, 21% 1 to 2, 39% 3+ Control: 60% 0, 23% 1 to 2, 17% 3+ |
| Henderson, 2024 | Cox | Age, sex, race, ethnicity, comorbidities, vaccination and SES | Hospitalization | 0: 44% 1 to 2: 32% 3+: 24% |
| Hsu CK 2024 | PSM | Age, sex, ethnicty, lifestyle factors, and comorbidities. | Hospitalization or death | NR |
| Huh, 2024 | PSM | Age, sex, comorbidities, vaccination, infection date, and previous COVID-19 infections | Death | <2: 4.9% 2: 5.7% 3+: 88.8% |
| Jorda, 2025 | PSM | Age, sex, immunization, symptoms, education, income, and comorbidities. | Hospitalization Death | 2+ doses in last 6 months: 55% |
| Kabore, 2023 | PSM | Age, sex, region of residence, vaccination, and comorbidities | Hospitalization | 0 or 1: 56% 2: 5% 3: 21% 4+: 18% |
| Kim, 2023 | LR | Age, sex, vaccination and comorbidities | Death | Vaccinated: 96% |
| Kim, 2024 | PSM | Age, sex, vaccination and comorbidities | Death | Vaccinated: 95% |
| Kuo, 2025 | Cox | Age, sex, vaccination, risk factors for severe COVID-19, comorbidity index, outpatient visits, and recent hospital admission. | Hospitalization Death Hospitalization or death | Treated: 11.4% 0 to 1, 7.7% 2, and 81% 3+ Control: 7.7% 0 to 1, 11.1% 2, and 81% 3+ |
| Kwok, 2023 | LR | Age, sex, respiratory disease, eGFR, and number of comorbidities. | Hospitalization Death | Vaccinated: 0% |
| Lewnard, 2023 | Cox | Age, sex, days of symptoms, comorbidities, vaccination, BMI, and healthcare utilization. |  | Treated: 6.1% 0 to 1, 13.3% 2, 80.6% 3+ Control: 14.9% 0 to 1, 25% 2, 60.1% 3+ |
| Lin 2023 | Cox | Age, sex, race, comorbidities, vaccination. | Death Hospitalization or death | Treated: 75% Control: 50% |
| Low 2023 | PSM | Age, sex, days of illness, vaccination, and comorbidities. | Hospitalization | Vaccinated (2+): 98% |
| Lui 2023 | PSM | Age, sex, comorbidities, Charlson score, vaccination, and medication use. | Hospitalization Death Hospitalization or death | Vaccination (3+): 67% |
| Ma 2023 | PSM | Age, sex, comorbidities, previous hospitalizations, laboratory tests. | Hospitalization | Vaccination: 21% |
| Paraskevis, 2023 | LR | Age, previous infection, vaccination, time since last vaccination. | Hospitalization Death | Treated: 90% 2+ Controls: 85% 2+ |
| Park, 2022 | PSM | Age, sex, vaccination, treatments | Death | NR |
| Rajme-López, 2024 | PSM | Age, sex, BMI, and comorbidities. | Hospitalization or death | Vaccination: 93% |
| Rodriguez-Leal, 2025 | Cox | Sex, age, immunosuppression, recent hospitalization, Charlson index, dialysis, hypertension, overweight, vaccination, previous COVID-19, and days of symptoms. | Hospitalization or death | 2 doses: 10% 3 doses: 67% 4 doses:16% |
| Saheb Sharif-Askari, 204 | Cox | Age, sex, race/ethnicity, comorbidities, vaccination. | Hospitalization | 2+ doses: 45% treated, 48% control |
| Schilling, 2024 | RCT | NA | Hospitalization | 2+ doses: 45% treated, 61% controls |
| Schwartz, 2023 | PSM | Age, sex, vaccination, comorbidities, long term care residence, and risk level. | Hospitalization or death Death | 2+ doses: 93% |
| Shah, 2023 | Cox | Age, sex, race/ethnicity, social vulnerabilities, comorbidities, vaccination, previous infection | Hospitalization | Treated: 15% 0, 19% 2, 60% 3+ Control: 28% 0, 23% 2, 42% 3+ |
| Shiau, 2024 | PSM | Age, sex, race/ethnicity, smoking, comorbidities, BMI and FEV1 predicted | Hospitalization or death | NR |
| Tsai 2023 | PSM | Age, sex, race, comorbitities, obesity | Hospitalization Death Hospitalization or death | NR |
| Van Heer, 2023 | LR | Sex, vaccination, previous hospitalization, and socioeconomic status |  | 1-2 doses: 6% 3 doses: 24% 4 doses: 70% |
| Wai, 2023 | Cox | Age, sex, previous health, ethnicity, socioeconomic status, comorbidities. |  | NR |
| Wee, 2023 | LR | Age, sex, ethnicity, housing, comorbidities, vaccination, previous infections. |  | 3+ doses: 95% |
| Wei 2025 | PSM | Age, sex, comorbidities, vaccination, medications, date, residence, healthcare utilization |  | 2+ doses: 85% |
| Wong 2022 | PSM | Age, sex, date, comorbidities, vaccination |  | 2+ doses: 33% |
| Wu JY 2023b | PSM | Age, sex, race, body mass index, comorbidities, labs |  | NR |
| Xie 2023 | PSM | Age, sex, race, deprivation, comorbidities, vaccination, medications, healthcare utilization |  | 0 doses: 76763 1-2 doses: 84620 2+ doses: 94905 |
| Yan, 2024 | PSM | Exact matching (age, vaccination, Charlson) and propensity score matching (sex, race, comorbidities) |  | 0 to 1 doses: 16% 2 doses: 23% 3+ doses: 61% |
| Yip 2024 | PSM | Age, sex, comorbidities, vaccination, hospitalizations |  | 2+ doses: 56% |

IPCW = inverse probability of censoring weights; PSM = propensity score matching; LR = logistic regression; IPTW = inverse probability treatment weighting; Cox = Cox regression

**Appendix Table 3**. Adapted Newcastle – Ottawa assessment scale for cohort studies.

| **DOMAIN** | **EXPANATION** | **CRITERIA** | **Possible maximum # of stars** |
| --- | --- | --- | --- |
| **Selection** | *Representativeness of exposed cohort* | **One star** = The cohort was truly representative of the average population of interest, i.e. community-dwelling adults with COVID-19 without further selection  **One star** = The cohort was somewhat representative of the average population of interest No star = selected group of users, e.g. nurses, volunteers etc.  No star = no description of the derivation of the cohort | **4** |
|  | *Selection of the non-exposed cohort* | **One star** = Drawn from the same community of the exposed cohort  No star = Drawn from a different source or no description |  |
|  | *Ascertainment of exposure* | **One star** = secure record (electronic healthcare records) **One star** = structured interview No star = written self-report or no description |  |
|  | *Demonstration that outcome of interest not present at start of study* | **One star** = yes, i.e. participants with the outcome were excluded from study No star = no |  |
| **Comparability** | *Comparability of cohorts on the basis of design or analysis* | **One star** = yes, i.e. study controls for at least age **One star** = plus another factor (e.g. comorbidity) | **2** |
| **Outcome** | *Assessment of outcome* | **One star** = independent blind assessment **One star** = record linkage No star = none of the above | **3** |
|  | Was follow-up long enough for outcomes to occur? | **One star** = yes, at least 30 days of follow-up No star = less than 30 days of follow-up |  |
|  | Adequacy of follow-up of cohorts | **One star** = complete follow-up (all subjects accounted for) or at least 80% (lost to follow-up unlikely to introduce bias) or random loss No star = follow-up below the treshold or no adequate description |  |

**Appendix Table 4**. Quality assessment of included studies according to NOS and AHRQ.

|  |  | **Newcastle Ottawa scale (NOS) assessment** | | | | **AHRQ assessment** |
| --- | --- | --- | --- | --- | --- | --- |
|  |  | **Selection** | **Comparability** | **Outcome/Exposure** | **Overall** |  |
| **Author,Year** | **PMID** | **(#) stars** | **(#) stars** | **(#) stars** | **(#) stars** |  |
| Aggarwal, 2023 | 36780912 | 4 | 1 | 3 | 9 | Good |
| Aggarwal, 2024 | 38500962 | 4 | 1 | 3 | 9 | Good |
| Al-Obaidi, 2023 | 36898600 | 4 | 1 | 3 | 9 | Good |
| Arbel, 2022 | 36001529 | 4 | 1 | 3 | 9 | Good |
| Bajema, 2023 | 37276589 | 4 | 1 | 3 | 9 | Good |
| Bhatia, 2025 | 39823513 | 4 | 1 | 3 | 9 | Good |
| Butt, 2024 | 37711076 | 4 | 1 | 3 | 9 | Good |
| Cegelon, 2023 | 37242504 | 3 | 1 | 2 | 7 | Fair |
| Cullen, 2024 | 39649410 | 4 | 1 | 3 | 9 | Good |
| Dormuth, 2023 | 37782496 | 3 | 1 | 3 | 8 | Fair |
| Dryden-Peterson 2023 | 36508742 | 3 | 1 | 3 | 8 | Fair |
| Evans 2023 | 36773891 | 4 | 1 | 3 | 9 | Good |
| Faust, 2023 | 37387690 | 3 | 1 | 3 | 8 | Fair |
| Ganatra, 2023 | 35986628 | 3 | 1 | 3 | 8 | Fair |
| Hansen, 2023 | 37398261 | 4 | 1 | 3 | 9 | Good |
| Henderson, 2024 | 38380946 | 4 | 1 | 3 | 9 | Good |
| Hsu CK 2024 | 38568820 | 4 | 1 | 3 | 9 | Good |
| Huh, 2024 | 39497563 | 4 | 1 | 3 | 9 | Good |
| Jorda, 2025 | 39505067 | 4 | 1 | 3 | 9 | Good |
| Kabore, 2023 | 37149726 | 4 | 1 | 3 | 9 | Good |
| Kim, 2023 | 37431541 | 4 | 1 | 3 | 9 | Good |
| Kim, 2024 | 38621766 | 4 | 1 | 3 | 9 | Good |
| Kuo, 2025 | 40988922 | 4 | 1 | 3 | 9 | Good |
| Kwok, 2023 | 36992319 | 3 | 1 | 3 | 8 | Fair |
| Lewnard, 2023 | 36933565 | 4 | 1 | 3 | 9 | Good |
| Lin 2023 | 37733342 | 4 | 1 | 3 | 9 | Good |
| Low 2023 | 37567557 | 4 | 1 | 3 | 9 | Good |
| Lui 2023 | 37204790 | 3 | 1 | 3 | 8 | Fair |
| Ma 2023 | 37103932 | 3 | 1 | 3 | 8 | Fair |
| Paraskevis, 2023 | 37565522 | 3 | 1 | 3 | 8 | Fair |
| Park, 2022 | 36617550 | 3 | 1 | 1 | 6 | Poor |
| Rajme-López, 2024 | 38545451 | 3 | 1 | 3 | 8 | Fair |
| Rodriguez-Leal, 2025 | 40412101 | 3 | 1 | 3 | 8 | Fair |
| Saheb Sharif-Askari, 204 | 39472619 | 4 | 1 | 3 | 9 | Good |
| Schwartz, 2023 | 36781188 | 4 | 1 | 3 | 9 | Good |
| Shah, 2023 | 36454693 | 4 | 1 | 3 | 9 | Good |
| Shiau, 2024 | 38702925 | 3 | 1 | 3 | 8 | Fair |
| Tsai 2023 | 37322616 | 3 | 1 | 3 | 8 | Fair |
| Van Heer, 2023 | 37927380 | 3 | 1 | 3 | 8 | Fair |
| Wai, 2023 | 36212676 | 4 | 1 | 3 | 9 | Good |
| Wee, 2023 | 37331509 | 3 | 1 | 3 | 8 | Fair |
| Wei 2025 | 39964106 | 4 | 1 | 3 | 9 | Good |
| Wong 2022 | 36216007 | 4 | 1 | 3 | 9 | Good |
| Wu JY 2023b | 37769748 | 3 | 1 | 3 | 8 | Fair |
| Xie 2023 | 37041016 | 3 | 1 | 3 | 8 | Fair |
| Yan, 2024 | 38864601 | 3 | 1 | 3 | 8 | Fair |
| Yip 2024 | 36031408 | 4 | 1 | 3 | 9 | Good |

Appendix Table 5. Definitions of immunocompromised used by individual studies. This table provides a summary, for a more granular explanation typically covering 2 pages or more for each study readers should review the appendices noted in the table.

| **Study** | **Definition** |
| --- | --- |
| **Any hospitalization** |  |
| Aggarwal, 2023 | Appendix 1, pg 10: Use of any of the following medications: TNF-alpha inhibitors, azathioprine, mycophenolate, systemic corticosteroids, alemtuzumab, azathioprine plus mTORi or calcineurin inhibitor, belatacept, eculizumab, rituximab, cyclophosphamide, thymoglobulin, calcineurin inhibitor alone and any of over 20 chemotherapy agents. |
| Kabore, 2023 | Appendix B: Leukemia, lymphoma, multiple myeloma, other hematologic cancer, solid organ transplant, and use of anti-B cell therapy, systemic corticosteroids, cyclophosphamide |
| Shah, 2023 | Suppl Table 2: Immunocompromise was defined using ICD-10 codes (adapted from https:// academic.oup.com/cid/article/73/11/e4353/6060064), primarily organ or stem cell transplant, immunodeficiency, connective tissue disorder) or immunocompromising medication (adapted from https://www.atsjournals.org/doi/10.1513/ AnnalsATS.201507-415BC) including chemotherapy, chronic steroid use, HIV, or radiation therapy prescribed during the past 6 months. |
| Wee, 2023 | Suppl MMC3: Presence of solid malignancy, hematologic malignancy, rheumatologic or inflammatory disorders, other intrinsic immune condition or immunodeficiency, or organ or stem cell transplant. |
| **Any hospitalization or death** |  |
| Dormuth, 2023 | Appendix 1, eTable 1: solid organ transplant, malignant hematologic condition, stem cell transplant, anti-CD20 agents, severe primary immunodeficiency, cancer treatment, significantly immunosuppressive drugs, advanced HIV, moderate primary immunodeficiency, renal conditions. |
| Al-Obaidi, 2023 | Table 3: Immunocompromised, status includes malignancy, lymphoproliferative disorders, and hematopoietic stem cell transplants, solid organ transplants, and patients with HIV. |
| Bajema, 2023 | Supp Table 3: Use of one of over 200 immunosuppressant drugs, largely chemotherapeutic agents |
| Lin, 2023 | eTable 2: Human immunodeficiency virus, pneumocystosis, malignant neoplasm associated with transplanted organ, other malignant immunoproliferative diseases; full list at https://www.qualityindicators.ahrq.gov/Downloads/Modules/PSI/V60-ICD10/TechSpecs/PSI_Appendix_I.pdf |
| Rodriguez-Leal, 2025 | Page 3: Solid or hematological transplant, renal replacement therapy, primary immunosuppression, active treatment with myelotoxic drugs, onco-hematological patients with either neutropenia or lymphopenia, human immunodeficiency virus infection with adult immunodepression syndrome stage or immunosuppressive treatment with corticosteroids, methotrexate,  or biologic drugs such as rituximab) |
| Yan, 2024 | Not reported in manuscript or supplemental materials |

Appendix Figure 1. Forest plots for all patients, all-cause mortality and COVID-specific mortality

| All-cause mortality   | COVID-specific mortality   |
| --- | --- |

Appendix Figure 2. Forest plots for all patients, all-cause hospitalization and COVID-specific hospitalization

| All-cause hospitalization   | COVID-specific hospitalization   |
| --- | --- |

Appendix Figure 3. Forest plots for any mortality, any hospitalization and composite of mortality or hospitalization stratified by age

| Mortality by age    Mortality or hospitalization by age   | Hospitalization by age   |
| --- | --- |

Appendix Figure 4. Forest plots for any mortality, any hospitalization and composite of mortality or hospitalization stratified by early vs late omicron

| Mortality by early vs late omicron    Mortality or hospitalization by early vs late omicron   | Hospitalization by early vs late omicron   |
| --- | --- |

Appendix Figure 5. Forest plots for any mortality, any hospitalization and composite of mortality or hospitalization stratified by immunocompromise

| Mortality by immunocompromise    Mortality or hospitalization by immunocompromise   | Hospitalization by immunocompromise   |
| --- | --- |

Appendix Figure 6. Forest plots for any mortality, any hospitalization and composite of mortality or hospitalization stratified by low vs high risk

| Mortality by low vs high risk    Mortality or hospitalization by low vs high risk   | Hospitalization by low vs high risk   |
| --- | --- |

Appendix Figure 7. Forest plots for any mortality, any hospitalization and composite of mortality or hospitalization stratified by PSM vs other method

| Mortality by PSM vs other method   | Hospitalization by PSM vs other method   | Mortality or hospitalization by PSM vs other method   |
| --- | --- | --- |

Appendix Figure 8. Forest plots for any mortality, any hospitalization and composite of mortality or hospitalization stratified by vaccination

| Mortality by vaccination  Mortality or hospitalization by vaccination   | Hospitalization by vaccination   |
| --- | --- |

Appendix Figure 9. Scatter plots with fitted regression lines and 95% Cis for hospitalization rate in the control group versus relative risk and mortality rate in the control group versus relative risk.

Hospitalization rate in control groups versus relative risk

Mortality rate in control groups versus relative risk

Appendix Figure 10. Funnel plots for outcomes of mortality, hospitalization and composite of mortality and hospitalization

| Mortality   | Hospitalization   |
| --- | --- |
| Mortality or hospitalization   |  |
